# Sterically Stabilized Homoleptic Copper and Gold Allyl Complexes

**DOI:** 10.1021/acs.organomet.5c00460

**Published:** 2026-02-20

**Authors:** Dillon Button-Jennings, Nathan D. Schley, Timothy P. Hanusa

**Affiliations:** Department of Chemistry, 5718 Vanderbilt University, VU Station B #351822, Nashville, Tennessee 37235, United States

## Abstract

Well-defined allyl derivatives of the coinage metals remain rare, often owing to their thermal and redox sensitivity. We report the isolation of a potassium bis­(allyl)­cuprate, [KCuA′_2_] (A′ = 1,3-(SiMe_3_)_2_C_3_H_3_), prepared with either solution or mechanochemical methods, which is stable at room temperature in both the solid state and solution. In the solid state, it crystallizes as a dimer ([{KCuA′_2_}_2_]), featuring a planar K_2_Cu_2_ core and η^1^-bound allyl ligands. In neat hexanes, [{KCuA′_2_}_2_] enables conjugate (1,4)-allylation of α,β-unsaturated ketones. It does not transmetallate when treated with lithium or sodium salts but converts to the crystallographically authenticated, but less stable neutral tetramer, [{CuA′}_4_], which possesses μ-η:^1^η^2^ allyl bridges and a puckered Cu_4_ ring. Gold analogs of both the anionic and neutral complexes have also been prepared ([{KAuA′_2_}_2_] and [{AuA′}_4_], respectively) and are structurally similar to their copper counterparts. However, the potassium bis­(allyl)­aurate does not perform the same addition chemistry as its copper analogue. DFT calculations on the unsubstituted di­(allyl) cuprates (i.e., [{(Li,K)­Cu­(C_3_H_5_)_2_}_2_]) suggest that both the trimethylsilyl groups and the substitution of Li^+^ with K^+^ influence the aggregation of potassium bis­(allyl)­cuprate. These findings provide structural and reactive benchmarks for coinage-metal allyl complexes.

## Graphical abstract

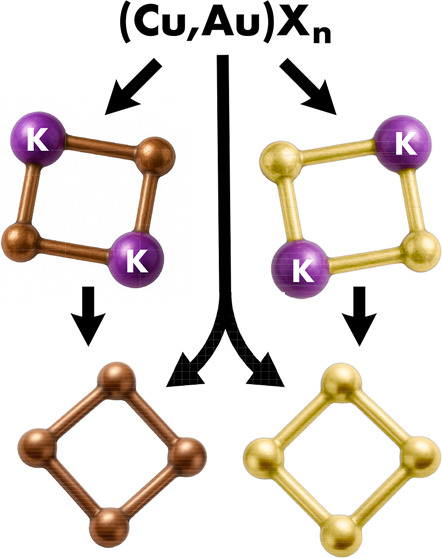

## Introduction

Since the mid-20th century, organocopper­(I) species (CuR, CuR_2_^–^, and the metal organocuprates MCuR_2_ and MCu­(X)­R (i.e., homocuprates and heterocuprates, respectively)) have become indispensable reagents for a range of organic transformations, including conjugate additions, cross-coupling reactions, and regioselective epoxide/aziridine ring openings.^1^ The composition of the reagents used in reactions is often more complex than their empirical formulas would suggest. The lithium organocuprates (LiCuR_2_, i.e., the Gilman reagents), for example, give their best rates and yields only as aggregated contact ion pairs (e.g., Li_2_Cu_2_R_4_, LiCu_4_Ph_6_^–^) in low-donor solvents (toluene, CH_2_Cl_2_, Et_2_O, Me_2_S, etc.);^2^ in highly polar or coordinating solvents such as THF, or when the lithium is sequestered in a crown ether,^3^ the resulting monomeric species display little to no reactivity. Hence, the structures of the aggregated species are of considerable interest and have been studied using NMR methods,^2c^ ^,^ ^4^ mass spectroscopy,^5^ and when possible, X-ray crystallography, which provides the most detailed view of their geometries.

A key question has been how closely structures in solution match those in the solid state, and there has often been good correlation between the two forms of the cuprates.^6^ With the more weakly coordinating Et_2_O or Me_2_S bases, dimeric and higher order structures have been confirmed (e.g., Li_2_Cu_2_Ph_4_(OEt_2_)_3_,^7^ [Li_2_Cu_2_(CH_2_SiMe_3_)_4_(L_*x*_)] (L_*x*_ = (OEt_2_)_3_, SMe_2_)_2_,^8^ Li_2_Cu_2_Ph_4_(SMe_2_)_3_,^9^ and ([Li_2_Cu_3_Ph_6_]_2_[Li_4_Cl_2_(Et_2_O)_10_][Bibr ref10]). In contrast, the stronger base THF can lead to the formation of monocopper species, as found in [Li­(THF)_4_]­[Cu­(C­(SiMe_3_)_3_)_2_].^11^ To be sure, the use of chelating^12^ or phosphine^13^ ligands can override these general trends, and how the organocuprates of the heavier alkali metals (Na^+^, K^+^) behave both chemically and structurally is far less studied.^14^ However, as with Li^+^, crown ethers and cryptands are found to create contact ion pairs in the solid state with K^+^, as occurs in [K­(THF)_2_(18-C-6)]­[Cu­(—C≡C—Ph)_2_][Bibr ref15] and [K[2.2.2]­crypt]­[Cu­(Mes)_2_].^16^

Understanding the correlations between the solid-state and solution structures of organocopper (I) nucleophiles is especially important when they exhibit limited solution stability and, in some cases, erratic reactivity.^17^ A poster child for these challenges is lithium diallylcuprate, Li­[Cu­(C_3_H_5_)_2_]. It exhibits many of the characteristic reactivity patterns of other lithium diorganocuprates,^18^ and it can facilitate conjugate allylation in complex molecule synthesis (such as forming stereodefined C–C bonds in natural product fragments).^19^ Yet it also displays high enough reactivity that in some cases it behaves more like a Grignard reagent, giving significant (or even dominant) 1,2-addition products rather than following the usual 1,4-(conjugate) pathway. Although such anomalous additions have sometimes been attributed to impurities in the reagents used to form the lithium diallylcuprate,^18c,20^ this only underscores the reagent’s sensitivity to its preparative sources. It can be formed by direct cupration of allyllithium (e.g., treatment of allyllithium with a Cu­(I) halide (often CuI) in Et_2_O/THF at −78 °C), and the solution used immediately. Even so, Li­[Cu­(C_3_H_5_)_2_] displays extreme thermal sensitivity; for example, a sample prepared at −105 °C and then transferred to an NMR spectrometer already showed decomposition products (e.g., 1,5-hexadiene, from Wurtz coupling) at −95 °C.^21^

Not surprisingly, no crystal structure of Li­[Cu­(C_3_H_5_)_2_] is known, and (allyl)copper structures in general are relatively rare. In recent years, bulky *N*-heterocyclic carbenes have been used as a strategy to provide stability and isolability to Group 11 allyl complexes, and structurally verified copper examples have been synthesized in the form [(NHC)­Cu­(σ-allyl′)]; these neutral compounds, which differ in their properties from Gilman reagents, are used in a variety of organic transformations, some of which are catalytically enantioselective.^22^ We were interested in exploring how two modifications to standard approaches could advance (allyl)copper chemistry: (1) The use of trimethylsilyl-substituted allyls, which have greatly expanded the range of other transition metal allyl complexes;^23^ and (2) the potential offered by mechanochemical synthesis routes.^24^ Typically, mechanochemical reactions require minimal or no solvent, consume less energy, and proceed more rapidly than traditional solvent-based reactions. Mechanochemistry can profoundly influence reaction outcomes by altering kinetics and mechanisms^25^ enabling access to novel compounds with higher yields and faster reaction times than conventional methods.^26^ Although we did not exclude traditional solution methods from our study, our goal was to minimize solvent use that could negatively affect synthesis, especially with the redox-sensitive Group 11 metals.^27^ The changes in ligand and preparative methods proved effective: we report the first room-temperature stable, isolated example of an unsupported (allyl)copper reagent, [{KCuA′_2_}_2_] (A′ = 1,3-(SiMe_3_)_2_C_3_H_3_), along with its neutral homoleptic allyl counterpart ([{CuA′}_4_]), and for reactivity comparisons, the analogous gold allyl complexes (Figure 1).

**Figure 1:**
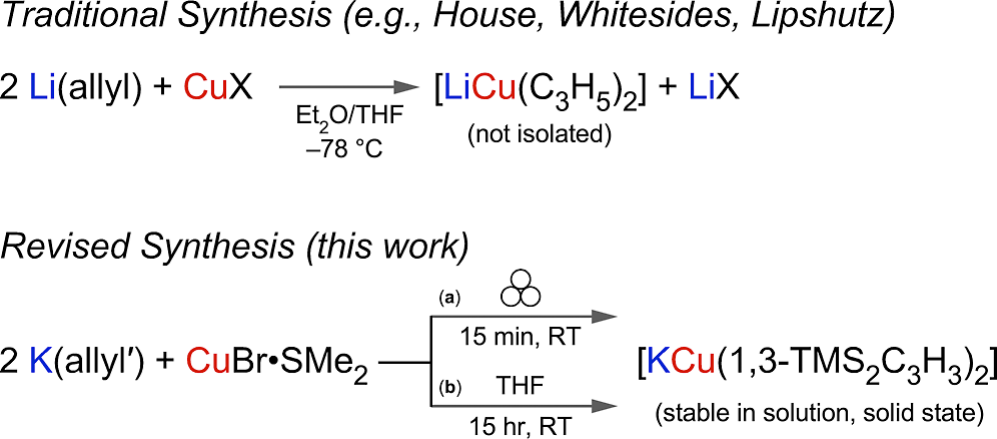
Conventional synthesis of lithium diallylcuprate from direct reaction of allyllithium with a Cu­(I) halide in an ether solution. The results presented here involve a substituted allyl (allyl′ = 1,3-(SiMe_3_)_2_C_3_H_3_), formed with either mechanochemical (a) or solution-based methods (b).

## Results and Discussion

### Synthesis

Our initial attempts to isolate an allyl copper complex involved the standard halide metathetical approach, but starting with the trimethylsilyl-substituted allyl ligand A′, which we believed would have a reasonable chance of forming thermally stable species. A solvent-free mechanochemical method was employed alongside conventional solution-based techniques.^23c^ ^,^ ^28^ However, the mechanochemical reaction of K­[A′] (as described below, the Li­[A′] salt does not work) with various Cu­(II) halides (CuCl_2_, CuBr_2_, CuI_2_) or phosphine adducts [CuCl_2_·(PR_3_)_0.5_] (R = Bu, Ph) resulted in a redox process that produced elemental copper and the Wurtz-like coupling product of two neutral radicals from the starting allyl anions, i.e., {A′}_2_ (1,3,4,6-tetrakis­(trimethylsilyl)­hexa-1,5-diene) (Table 1, eq 1). As mentioned earlier, the corresponding coupled compound from the parent allyl (i.e., 1,5-hexadiene) is also a common decomposition product of the unsubstituted reagent [LiCu­(C_3_H_5_)_2_].^21^

**Table 1:**
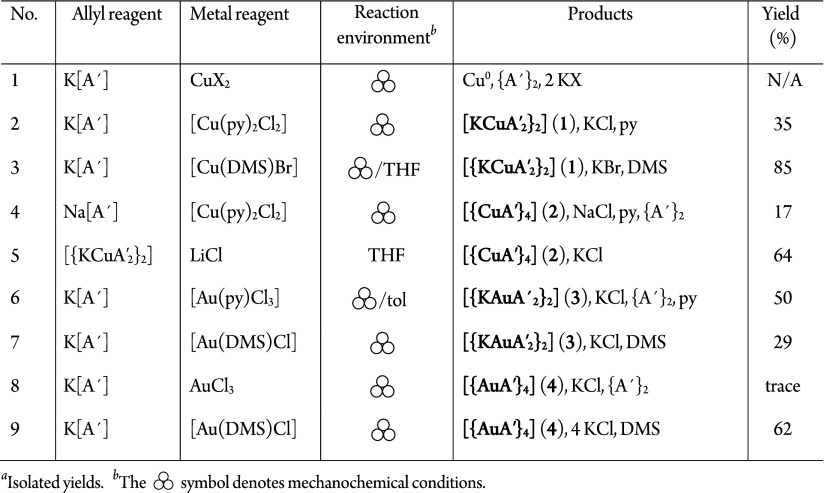
Reactions to Form Copper and Gold Allyl Complexes

An analogous undesired redox reaction was encountered during the mechanochemical synthesis of the nickel allyl complex [NiA′_2_],^29^ which was mitigated by using a pyridine solvate of NiCl_2_.^30^ We explored this potential synthetic approach using [Cu­(py)_2_Cl_2_][Bibr ref31] as the copper source with K­[A′] in both THF and toluene solutions. Unfortunately, the reaction in either solvent again produced elemental copper and {A′}_2_.

In contrast, ball milling a 2:1 mixture of K­[A′] and [Cu­(py)_2_Cl_2_] for 15 min, followed by hexane extraction of the ground solid, produced a tan oil that could be recrystallized from hexanes to yield colorless crystals. A single-crystal X-ray structure determination confirmed the solid as the potassium allyl cuprate, [{KCuA′_2_}_2_] (**1**) (Table 1, eq 2). Once it was clear that the product was a Cu­(I) species, higher yields (85%) were achieved by starting with a Cu­(I) precursor, the dimethylsulfide (DMS) adduct of CuBr (Table 1, eq 3) (Figure 2a). We subsequently found that mechanochemical activation is not essential when using [Cu­(DMS)­Br], as the reaction in THF also produces **1**, albeit with a slightly lower yield (64%). The allyl ligands in **1** exhibit fluxionality in solution, as shown by the symmetric (“π-bound”) set of resonances observed in the ^1^H NMR spectra in C_6_D_6_.

**Figure 2:**
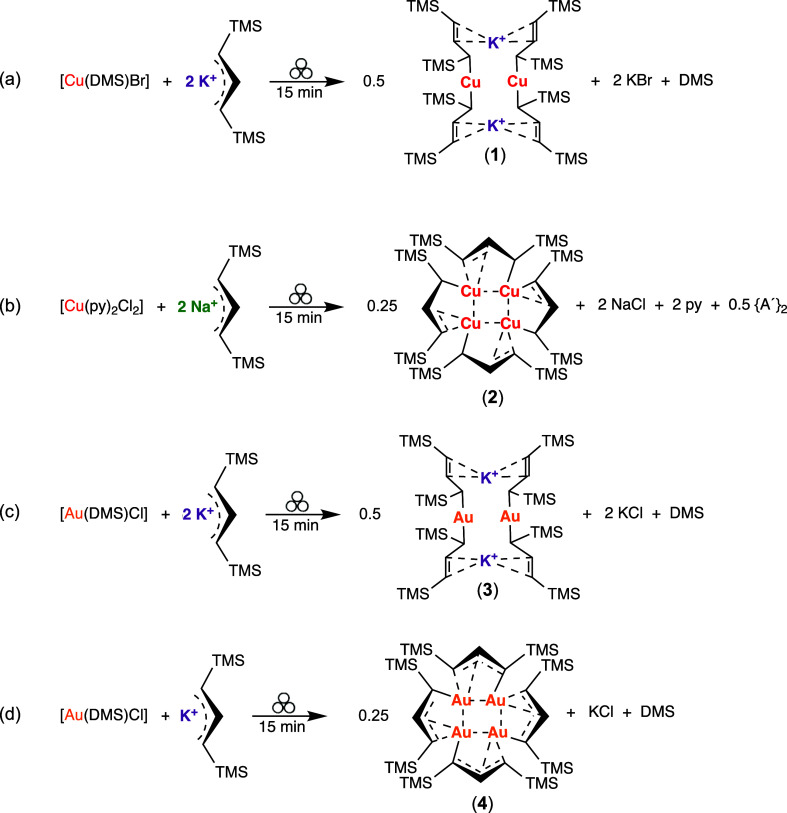
Synthesis routes to substituted allyl complexes: (a) [KCuA′_2_}_2_] (**1**); (b) [{CuA′}_4_] (**2**); (c) [KAuA′_2_}_2_] (**3**); (d) [{AuA′}_4_] (**4**).

We attempted to synthesize a lithium or sodium analog of **1** using reagents similar to those in Table 1, eq 2. Surprisingly, using Na­[A′] did not produce the expected [{NaCuA′_2_}_2_], but instead yielded the yellow neutral copper allyl [{CuA′}_4_] (**2**), which, to our knowledge, is the first reported isolated homoleptic copper allyl complex (Table 1, eq 4) (Figure 2b). The same product also forms with Li­[A′], or when attempting to exhange potassium for lithium by treatment with lithium salts (Table 1, eq 5). In solution, ^1^H NMR evidence shows that only one type of allyl ligand is present in **2** and, like **1**, it is fluxional, appearing as an η^3^-bound ligand. The compound can be handled under nitrogen for short periods of time at room temperature, but otherwise decomposes within a day, leaving a dark residue. It should be noted that the compound as initially obtained is not pure; as eq 2b in Figure 2 indicates, an accompanying byproduct of the synthesis of **2** is the substituted hexadiene {A′}_2_, actually formed in twice the molar amount as the tetrameric **2**. The hexadiene has similar solubility properties to **2** and cannot be effectively removed by solvent extraction. It is possible to cool solutions of **2** in hexanes to −40 °C and precipitate small quantities (mg) of crystalline **2**, which are suitable for single crystal X-ray crystallography. This is admittedly not a method for producing gram (or even tens of mg) of pure product, but despite trying various precursors, including a Cu­(I)-containing species ([Cu­(DMS)­Br]), which could potentially avoid the redox chemistry that produces {A′}_2_, to date we have not found a more efficient synthetic route.

Efforts to isolate silver allyl complexes were conducted in the absence of light. Reactions with K­[A′] involved various AgX salts and phosphine adducts (X = BF_4_, triflate, Cl, Br, I, Cl·PPh_3_, Cl·P­(nBu)_3_, Cl·(PPh_3_)_2_(py)), but all yielded only the hexadiene {A′}_2_ and films of Ag.

Given the sometimes parallel chemistry between Cu and Au complexes within a ligand environment, we attempted to prepare the corresponding gold complexes using similar synthetic methods. The positive reactivity observed with pyridine-solvated copper halides suggested that the pyridine gold (III) chloride [Au­(py)­Cl_3_] could be an effective gold source. The potassium allyl metathesis reactions with copper were in fact replicated; i.e., treatment of [Au­(py)­Cl_3_] with K­[A′] produces the bis­(allyl) aurate (**3**) as colorless crystals (Table 1, eq 6). Similar to the cuprate, the aurate can be synthesized through mechanochemistry or solution methods. Improved purity (i.e., without the formation of {A′}_2_) was obtained from the Au­(I) precursor [Au­(DMS)­Cl] (Table 1, eq 7) (Figure 2c). Unlike the NMR resonances in **1**, the aurate shows σ-bound allyl ligands in solution; e.g., separate resonances are observed for the two TMS groups.

A neutral gold allyl complex can be synthesized in several ways, not all of which correlate with the copper chemistry. Unlike the formation of **2**, grinding or dissolving [Au­(py)­Cl_3_] with Na­[A′] does not produce a gold complex, but results in the reduction of the gold. Even though unsolvated AuCl_3_ and K­[A′] also react in toluene to form gold films, using the same reagents in a mechanochemical context results in controlled reduction and the trace formation of pale yellow [AuA′]_4_ (**4**), along with substantial amounts of {A′}_2_ (Table 1, eq 8). Better results were obtained with the same reagents that produced **3**, specifically, the Au­(I) salt [Au­(DMS)­Cl] and K­[A′], used in a 1:1 ratio (Table 1, eq 9) (Figure 2d). The solution ^1^H NMR spectrum of **4** displays resonances consistent with π-bound allyl ligands, as evidenced by single resonances for the two TMS groups in both ^1^H and ^13^C NMR spectra.

### Crystallographic Results

#### [{KCuA′_2_}_2_] (**1**)

Crystals of **1** were obtained through slow evaporation from hexanes. In the solid state, **1** is constructed around a K_2_Cu_2_ ring with η^1^-bound allyl ligands (σ-A′, Figure 3). The formation of cyclic dimeric aggregates is known in lithium diorganocuprates,^32^ as is the nearly linear (175.6°) C–Cu–C′ arrangement of the allyls around the copper atoms in [CuX_2_]^−^ systems.^33^ The molecule has a crystallographically imposed inversion center, so only half of it is unique, and the K_2_Cu_2_ ring is necessarily flat.

**Figure 3:**
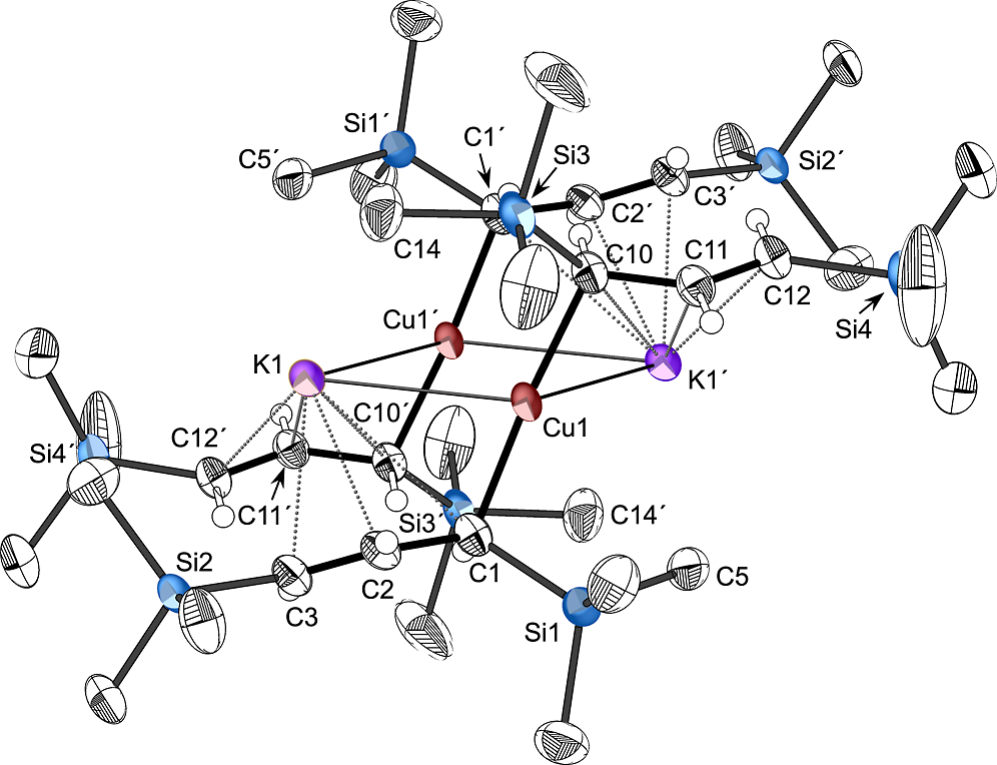
Thermal ellipsoid plot (50% level) of [{KCuA′_2_}_2_] (**1**). For clarity, hydrogens (assigned an arbitrary radius) have been removed from the trimethylsilyl groups. Selected bond distances (Å) and angles (°): Cu1–C1, 1.9602(15); Cu1–C10, 1.9647(15); C1–C2, 1.468(2); C2–C3, 1.353(2); K1···C1, 3.3428(16); K1···C2, 3.1732(16); K1···C3, 3.0323(15); K1···C10′, 3.1551(15); K1···C11′, 3.1862(15); K1···C12′, 3.0575(16); Cu1···K1, 3.2314(4); Cu1···K1′, 3.3072(4), C1–Cu1–C10, 175.56(6); K1–Cu1–K1′, 103.873(9); Cu1–K1–Cu1′, 76.127(9).

The Cu–C­(allyl) distances (1.962(2) Å, ave.) are similar to those found in the few known copper allyl complexes,^22a–22c^ and the C1–C2 and C2–C3 distances differ by 0.12 Å, indicating substantial electron localization into single and double bonds. The K^+^ ions are an integral part of the structure. Although the bonding between potassium and copper is clearly highly polar, the K–Cu distances (3.23, 3.30 Å) fall within the sum of the covalent radii for each element (3.35 Å).^34^ There are few other structurally confirmed K/Cu molecules for useful comparison with this framework, but one is the potassium salt of the bis­(anthracene)­dicuprate dianion, [K­(THF)_2_]_2_[{Cu­(9,10-η^2^-anthracene)}_2_].^35^ In this case, the two anthracenes bind the coppers together, and η^6^-contacts with the end aromatic rings support the potassium ions. The center (KCu)_2_ ring, although flat, is more distorted than in **1**, and the Cu···K distances are less similar (3.38, 3.66 Å) than the 0.08 Å difference in **1**. Another complex that could be compared to **1** is [({SiN^Dipp^}­Al)_2_Cu]­K, whose K^+^ counterion is supported by η^6^-contacts with the phenyl rings of the Dipp ligand.^36^ The Cu–K distance of 3.01 Å is considerably shorter than in **1**, partially reflecting a lower coordination number for potassium.

Contacts between the K^+^ ions and the double-bonded carbons of the A′ ligands in **1** (3.03–3.19 Å) are well within the range for energetically significant cation–π interactions.^37^ Although unquestionably weaker, the K^+^···H­(Me) contacts (K1···C5, C14) at 3.45 and 3.42 Å are still shorter than the typical packing distance range (roughly >3.6–3.7 Å).^38^

#### [{CuA′}_4_] (**2**)

Crystals of compound **2** were obtained through cooling a hexanes solution to −40 °C. The X-ray single-crystal structure of **2** indicates that it exists as a tetramer in the solid state, assembled around a puckered Cu_4_ ring, with the metals bridged by the allyl ligands in a μ_2_-η:^1^η^2^-bonding mode (Figure 4). There is no crystallographic symmetry imposed on the molecule, although an approximate C_2_ axis exists perpendicular to the Cu_4_ ring. Tetracopper complexes are well established, but most examples exhibit linear metal arrangements.^39^ Rings are known in “dimer of dimer” complexes such as [Cu_2_(Ophen)_2_]_2_ and [Cu_2_(Obpy)_2_]_2_,^40^ but true intramolecular cyclic Cu_4_ rings, such as in **2**, are rarer. They are found in tetrameric mesityl copper (with a mean Cu–Cu′ distance of 2.42 Å),^41^ 2,4,6-triisopropylphenylcopper (Cu–Cu′ distance of 2.445(1) Å),^42^ and *o*-vinylphenylcopper (mean Cu–Cu′ distance of 2.40 Å),^43^ as well as in the set of heteroleptic aryl copper (I) thiolato complexes of formula {Cu_2_(SAr)­Mes}_2_ (Cu–Cu′ distance bridged by the thiolato groups are 2.82(7) and 2.86(7) Å; those bridged by the mesityl ligands are shorter, at 2.43(5) and 2.43(5) Å.)^44^ The Cu–Cu′ distances in the homoleptic **2** are actually somewhat similar to those of the heteroleptic thiolates, as they fall into two distinct groups: there are two adjacent Cu–Cu′ distances averaging 2.584(1) Å, and two other, averaging 2.826(1) Å. As a result, the cross-ring distances in **2** are notably unequal (Cu2···Cu4, 3.40 Å; Cu1···Cu3, 4.13 Å). The angle between the two Cu_3_ planes (Cu_2_/Cu_3_/Cu_4_, Cu_2_/Cu_1_/Cu_4_) is 157.6°.

**Figure 4:**
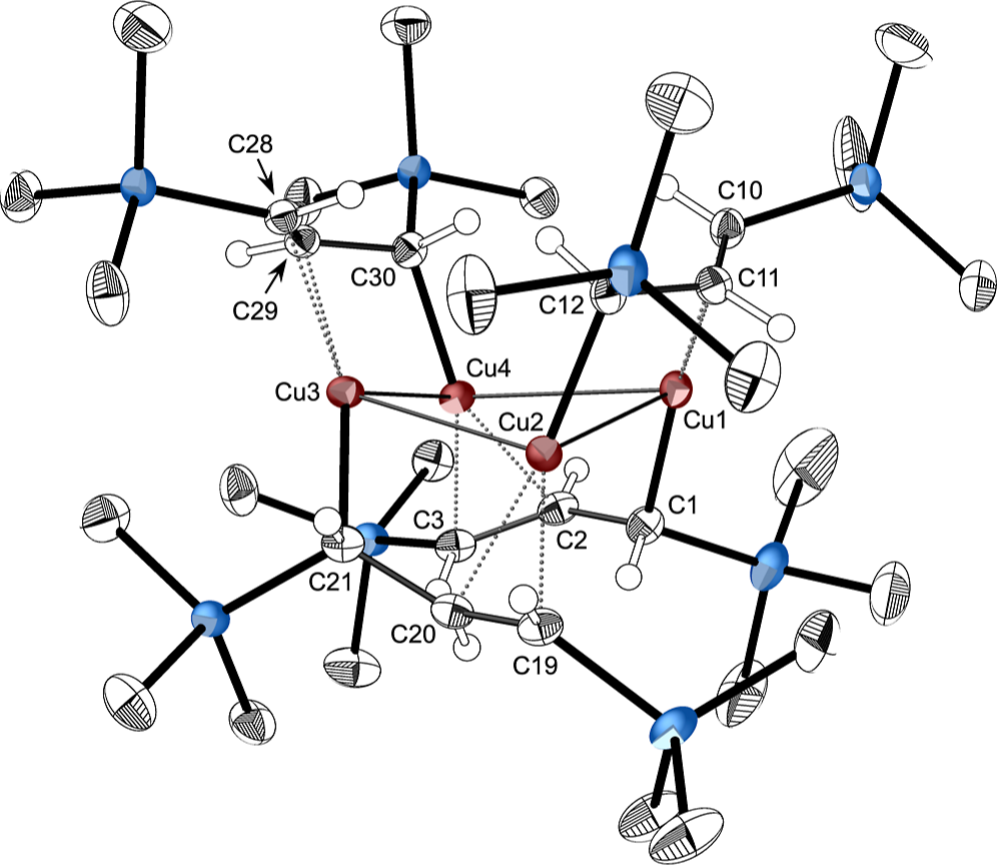
Thermal ellipsoid plot (40% level) of [{CuA′}_4_] (**2**). For clarity, hydrogens (assigned an arbitrary radius) have been removed from the trimethylsilyl groups. Selected bond distances (Å) and angles (°): C1–C2, 1.465(3); C2–C3, 1.384(3); C10–C11, 1.391(3); C11–C12, 1.448(3); C19–C20, 1.389(3); C20–C21, 1.466(3); C28–C29, 1.375(3); C29–C30, 1.472(3); Cu1–C1, 1.970(2); Cu1···C10, 2.082(2); Cu1···C11, 2.1522(18); Cu2–C12, 1.9772(19); Cu2···C19, 2.1331(19); Cu2···C20, 2.0752(19); Cu3–C21, 1.9919(19); Cu3···C28, 2.1834(18); Cu3···C29, 2.0765(18); Cu4–C30, 1.9808(19); Cu4···C2, 2.1326(19); Cu4···C3, 2.1106(19); Cu1–Cu2, 2.8153(4); Cu2–Cu3, 2.5814(4); Cu3–Cu4, 2.5867(4); Cu1–Cu4, 2.8369(4); Cu2–Cu1–Cu4, 73.881(10); Cu3–Cu2–Cu1, 99.661(12); Cu2–Cu3–Cu4, 82.187(12); Cu3–Cu4–Cu1, 98.978(12).

In contrast to **1**, the bridging allyl ligands in **2** display signs of some π-delocalization between single and double bonds, as the average bond lengths (1.463(6) and 1.385(6) Å, respectively) differ by only 0.078 Å. The Cu–C interactions are accordingly more complex than in **1**. Each copper atom forms a primary bond to carbon at a typical single-bond distance of 1.98 Å (ave.). There is a secondary interaction between each copper and the double bond of an adjacent ligand; for example, Cu2 bonds to C12 at 1.977(2) Å, then to the double-bonded carbons C19/C20 at a distance of 1.986 Å (midpoint of the C=C bond). The latter is a typical separation for Cu­(I)–olefin interactions (1.88–2.05 Å, 1.95 Å ave.).^45^

A point of some interest are the large number of interligand H···H′ distances that are that are ≤2.4 Å, i.e., less than the sum of the van der Waals radii for the hydrogen atom; the number serves as a proxy for the strength of dispersion forces.^46^ Although bulky groups can often serve as molecular stabilizers by providing dispersion energies,^23c^ ^,^ ^47^ the 24 such contacts in **2** (visualized in Figure S13) are not enough to guard it against facile decompostion.

#### [{KAuA′_2_}_2_] (**3**)

Crystals of **3** were obtained through slow evaporation from hexanes. Despite differences in radii and solution behavior, compounds **1** and **3** are isostructural in the solid state. The aurate **3** is built around a K_2_Au_2_ ring with a nearly linear (174.60(12)°) C–Au–C′ arrangement of the allyls around the gold (Figure 5). Similar to compound **1**, **3** has a crystallographically imposed inversion center, making only half unique, and the K_2_Au_2_ ring is flat.

**Figure 5:**
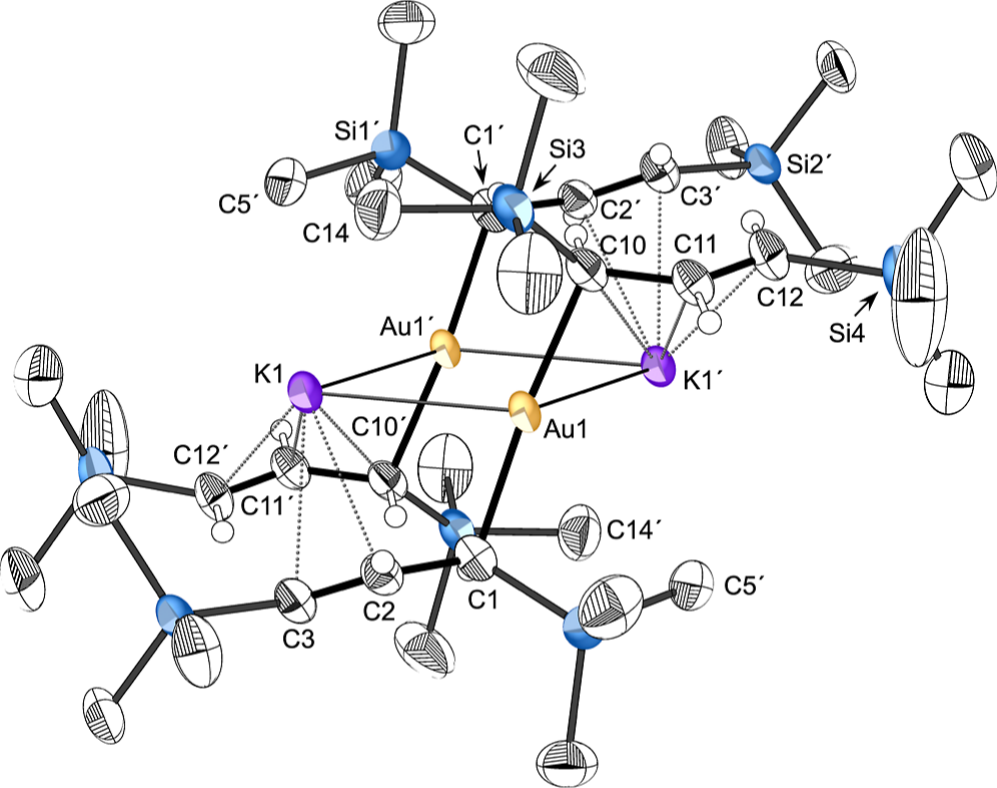
Thermal ellipsoid plot (40% level) of [{KAuA′_2_}_2_] (**3**). For clarity, hydrogens (assigned an arbitrary radius) have been removed from the trimethylsilyl groups. Selected bond distances (Å) and angles (°): Au1–C1, 2.118(3); Au1–C10, 2.117(3); C1–C2, 1.472(4) Å; C2–C3, 1.350(4); K1···C2, 3.219(3); K1···C3, 3.107(3); K1···C10, 3.207(3); K1···C11′, 3.238(3); K1···C12′, 3.111(3); Au1···K1, 3.2253(6); Au1···K1′, 3.2828(6), C1–Au1–C10, 174.60(12); K1···Au1···K1′, 105.098(14); Au1···K1···Au1′, 74.901(14).

The Au–C­(allyl) distances (2.12 Å, ave.) are typical for Au­(I)–C alkyl/aryl bonds,^48^ and the C1–C2 and C2–C3 distances differ by 0.12 Å, similar to **1**, indicating substantial electron localization into single and double bonds. Also like compound **1**, the K^+^ ions are integral to the structure. The K–Au distances (3.2253(6) and 3.2828(6) Å) are within the sum of the covalent radii for each element (3.39 Å),^34^ but are longer than the K–Au distance in the [({SiN^Dipp^}­Al)_2_Au]K complex (3.10 Å), whose K^+^ counterion is supported by η^6^-contacts with the phenyl rings of the Dipp ligand.^36^ Distances between the K^+^ ions and double-bonded carbons of the A′ ligands (ranging from 3.11–3.24 Å) are well within those expected for cation–π interactions.^37^ In contrast, the K^+^···H­(Me) distances are even longer than in the copper analog, making them of marginal importance. For example, the K1···C14 separation of 3.52 Å is potentially of some energetic significance,^38^ but it approaches the distance of the closest intermolecular contact (K1···C9″, 3.57 Å), and thus could be influenced by packing forces.

#### [{AuA′}_4_] (**4**)

Crystals of compound **4** were obtained through slow evaporation from hexanes. There are two crystallographically independent molecules of **4** in the asymmetric unit (space group *P*, *Z* = 4), but since they are similar, only one will be discussed here. In the solid state, **4**, like **2**, exists as a tetramer, arranged around a puckered Au_4_ ring, with the metals bridged again by allyl ligands in a μ_2_-η:^1^η^2^-bonding mode (Figure 6). This is one member of a large family of crystallographically confirmed gold rings, in which the Au atoms form a cyclic backbone supported by organic ligands ({AuR}_n_).^49^ The Au_4_ ring is more regular than that in **2**, with an average Au–Au′ distance of 3.106(4) Å (range = 3.08–3.13 Å), and the angle between the two Au_3_ planes is 171.4°. These are typical values for such rings, although direct comparisons with other ({AuR}_4_) species can be challenging, as the nature of the bridging ligands can influence the “soft” Au···Au′ separations. The structurally related tetragold (I) formamidinate cluster complex [{Au­(ArNC­(H)­NAr)}_4_] (Ar = C_6_H_4_-4-OMe), for example, exhibits an average Au–Au′ distance of 2.94 Å (range = 2.90–3.01 Å), which is imposed by the smaller bite angle of the formamidinate anion compared to [A′]^−^.^49b^

**Figure 6:**
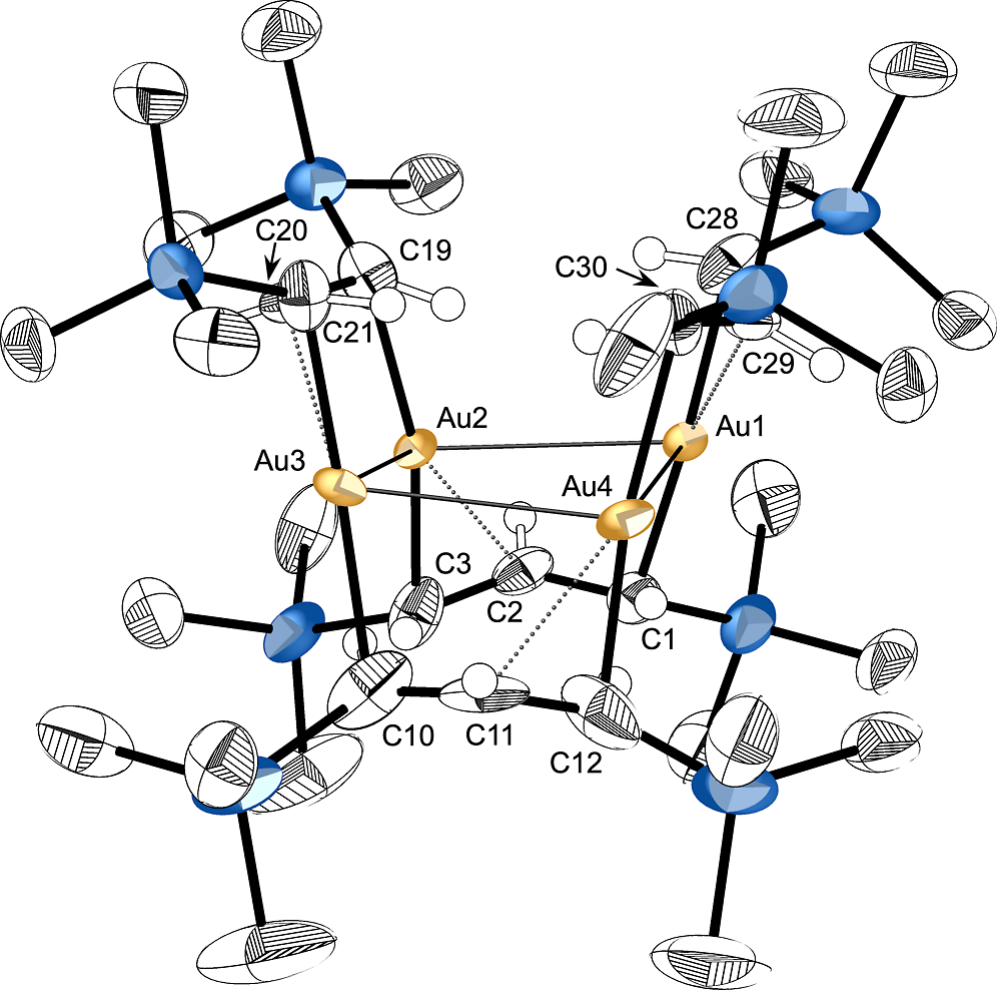
Thermal ellipsoid plot (33% level) of [{AuA′}_4_] (**4**). For clarity, hydrogens (assigned an arbitrary radius) have been removed from the trimethylsilyl groups. Selected bond distances (Å) and angles (°): C1–C2, 1.412(10); C2–C3, 1.391(11); C10–C11, 1.391(14); C11–C12, 1.376(13); C19–C20, 1.402(9); C20–C21, 1.402(10); C28–C29, 1.395(10); C29–C30, 1.398(11); Au1–C1, 2.209(11); Au1–C28, 2.124(9); Au1···C29, 2.335(8); Au2–C3, 2.083(9); Au2–C19, 2.215(8); Au2···C2, 2.282(6); Au3–C10, 2.222(11); Au3–C21, 2.109(9); Au3···C20, 2.344(7); Au4–C12, 2.093(11); Au4–C30, 2.192(9); Au4···C11, 2.423­(9); Au1···Au2, 3.0794(17); Au2···Au3, 3.108(2); Au3···Au4, 3.110(3); Au1···Au4, 3.127(2); Au2···Au1···Au4, 82.15(7); Au1···Au2···Au3, 97.88(6); Au2···Au3···Au4, 81.97(7); Au3···Au4···Au1, 96.84(8).

The delocalization of the π-electrons in the bridging allyl ligands in **2** is nearly complete in **4**, as the C···C′ bonds differ by only about 0.01 Å. The Au1–C1 bond is longer than Au2–C3 by 0.126 Å; as a result, Au2 and the central carbon of the allyl (C2) are separated by 2.28 Å, which could indicate a weak interaction.^50^ The Au1···C2 distance, in contrast, is at 2.89 Å, too far for meaningful interaction. These distortions, with delocalized ligands and unequal terminal atom distances to a metal, fit a pattern previously described as “slipped π”.^51^

### 1,4-Conjugate Addition Reactions

1,4-Conjugate addition of alkyl groups to α,β-unsaturated ketones is a key reaction for diorganocuprates.^1a,1c^ Not surprisingly, the use of lithium diallylcuprate has often proven to be unpredictable with these reactions, with varying results from different reports;^52^ the details of its preparation (copper source, etc.) contribute to this variability.^2d,18a,18c,20,53^ Although complex **1** possesses the [MCu­(allyl′)_2_] composition of [LiCu­(C_3_H_5_)_2_] and is a thermally stable, crystalline material, the addition of trimethylsilyl groups and especially the replacement of Li^+^ with K^+^ are changes that could interfere with the behavior in **1** in Gilman-like chemistry.

In a previous DFT computational study, it was found that cooperation between the two lithium atoms and copper in dimeric (LiCuMe_2_)_2_ was crucial for the 1,4-conjugate addition of the methyl group to acrolein; in contrast, the smaller LiCuMe_2_ could not perform the addition effectively, and in any case electrophilic assistance by the lithium atom played an essential role in the reaction.^54^ It is perhaps not surprising that the deliberate replacement of lithium with other alkali metals in Gilman chemistry is extremely rare. The only systematic study of which we are aware is from Bertz, who reported that various organosodium compounds (NaR) react with CuCN or CuBr·SMe_2_ in THF at low temperature (−50 °C) to give NaCuR_2_. These reagents displayed “typical cuprate reactivity,” although they were generally less reactive than their lithium analogs toward enones.^14^ Given this singular reference to a sodium analog of a Gilman reagent, it is not surprising that there are no reports of potassium diorganocuprates (KCuR_2_) being prepared and used as discrete reagents under standard cuprate conditions.

There are some known examples of substituted allyls or similar ligands used in Gilman reagents.^55^ However, none of these have the steric bulk of the trimethylsilyl-substituted A′, however, so its effect on reactions could be substantial (note that the relative van der Waals volume of [A′]^−^ (231 Å^3^) is four times that of the parent allyl anion, [C_3_H_5_]^−^ (57 Å^3^)).^56^

The alkane solubility provided by the TMS groups on **1** enabled us to study conjugate additions in hexanes, a solvent not normally used alone in standard Gilman chemistry.^57^ Adding 2-cyclohexenone to a solution of **1** in hexanes produced 3-(1,3-bis­(trimethylsilyl)­allyl)­cyclohexan-1-one in modest yield (34%), the result of 1,4-conjugate addition (Scheme 1a). Similarly, allowing 3-butenone to react with **1** generated 5,7-bis­(trimethylsilyl)­hept-6-en-2-one at a comparable yield (Scheme 1b). The reactions were conducted as proof-of-concept experiments; nonetheless, they yielded results comparable to or better than those obtained with sodium dibutyl cuprates or cyanocuprates.^14^ Bertz discovered that sodium reagents prepared from CuCN or CuBr·SMe_2_ yielded only trace amounts (1.6% and 0.6%, respectively) of the 1,4-addition product with 2-cyclohexenone at −78 °C, increasing to 30% and 12%, respectively, at −50 °C. Although limited, these comparisons suggest that switching from Na^+^ to K^+^ does not negatively affect reaction yield, although neither metal is as effective as Li^+^. It should be noted that the solvent choice has a strong impact on the reactions with **1**; changing from hexanes to THF decreased the yield of the corresponding ketone from 33%–34% to significantly poorer amounts (<10%). This loss of activity parallels the behavior of traditional Gilman reagents,^2^ and is likely related to disaggregation that occurs in highly polar media.

**Scheme 1:**
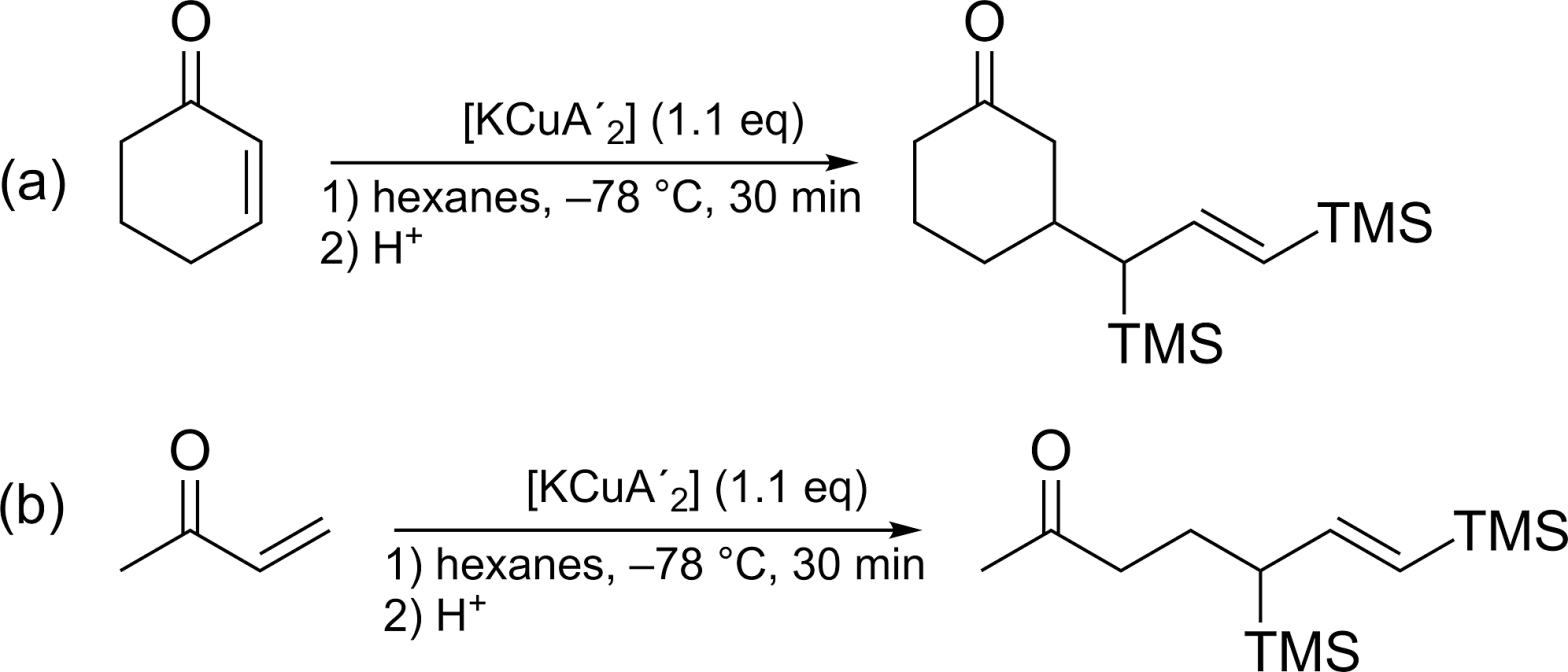
Reaction of **1** with (a) 2-Cyclohexenone; (b) 3-Butenone

In contrast to the cuprate, under the same conditions, the allyl aurate **3** did not promote the addition of A′ to α,β-unsaturated ketones in either a 1,2 or 1,4 fashion. It is known that gold (or silver) -ate species rarely react with a carbon electrophile to form new C–C bonds, a difference attributed to the accessibility of high-lying d-orbitals that directly participate in the nucleophilic reaction.^58^ We found that upon workup of these attempted addition reactions, **3** hydrolyzes to generate the tetragold complex **4** and HA′. Although not exactly parallel behavior, Kochi studied the hydrolytic stability of organoaurates in the context of ligand stabilization.^59^ Specifically, the dimethylaurate triphenylphosphine adduct ((CH_3_)_2_AuLi­[PPh_3_]) reacted with water to generate the neutral methylgold triphenylphosphine adduct ((CH_3_)­Au­[PPh_3_]). However, such behavior is not universal with aurates, as the pentamethyldiethylenetriamine (PDMTA) adduct ((CH_3_)_2_AuLi­(PMDT)) produced Au^0^ upon hydrolysis.

### Computational Results

There are several points of computational interest in **1**–**4**, such as the differences between the -ate and neutral species, the copper/gold pairings (**1**/**3**, **2**/**4**), and the presence of metal–metal bonding in **2** and **4**. As regards the latter, the M–M′ distances in **2** and **4** are consistent with d^10^–d^10^ metallophilic interactions,^60^ a phenomenon often linked with photoluminescent properties that depend on the ligand environment.^61^ An atom-in-molecules (AIM) analysis of **2** and **4** shows bond paths and bond critical points between the metal atoms, with electron densities indicative of weak metallophilic interactions (see Supporting Information 17–18).

Our focus here, however, is on the organocuprate **1**, specifically how the trimethylsilyl groups on the allyl ligands and the replacement of Li^+^ with K^+^ contribute to its structure compared to the classic Gilman reagent [LiCu­(C_3_H_5_)_2_]. Removing the TMS groups from **1** (i.e., to form [{KCu­(C_3_H_5_)_2_}_2_]), followed by geometry optimization (B3PW91-D3BJ/def2TZVP; no imposed symmetry^62^), results in a structure whose core resembles the original (Figure 7a). The allyl ligands remain localized (the difference between single/double bonds, e.g., C1–C2/C2–C3, is 0.12 Å, the same as in **1**), and the average Cu–C bond length (1.976 Å) closely matches the crystallographic value, within 0.02 Å. Potassium shows some overbinding to copper (ave. Cu–K = 3.13 Å (calc.); 3.27 Å (crystal), but not to the allyl ligand (K–C­(allyl) = 3.19 Å (calc.); 3.16 Å (crystal)). The calculated (CuK)_2_ ring is much closer to a square than in the crystal structure, with the difference between Cu–K–Cu′ and K–Cu–K′ angles being 2.7° (calc.) and 27.8° (crystal). A notable difference lies in the orientation of the two CuA′_2_ units relative to each other. In **1**, the twist angle between the two C–Cu–C′ vectors (the Cu–C–C′–Cu′ torsion angle) is small (3.6°); in [{KCu­(C_3_H_5_)_2_}_2_], the calculated twist is much greater (33.5°). Generally, the additional TMS groups in **1** cause greater distortion in the central (KCu)_2_ ring, but keep the two CuA′_2_ fragments closer together in space (cf. the Cu···Cu′ separation of 4.37 Å and 4.03 Å in [{KCu­(C_3_H_5_)_2_}_2_] and **1**, respectively).

**Figure 7:**
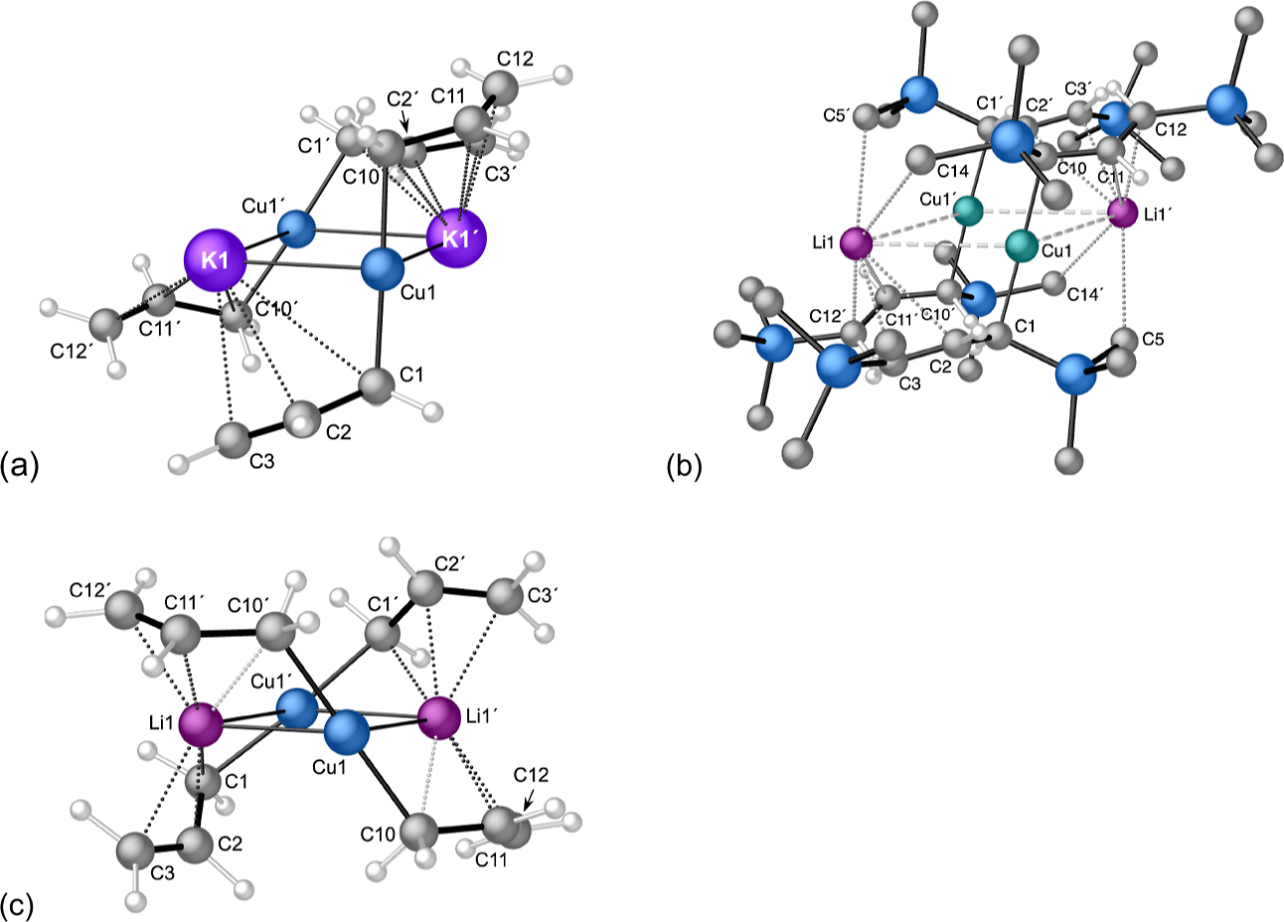
Geometry-optimized structures and selected distances (Å) (B3PW91-D3BJ/def2TZVP); for ease of comparison, the numbering scheme follows that in Figure 3, and for (a) and (b) the (′) designation now denotes those atoms that were related by inversion symmetry in **1**, but have shifted in the modified complexes ((c) has reoriented enough that a direct comparison is not feasible): (a) [{KCu­(C_3_H_5_)_2_}_2_]; Cu1–C1, 1.973 (Cu1′–C1′, 1.978); Cu1–C10, 1.973 (Cu1′–C10′, 1.978); C1–C2, 1.464 (C1′–C2′, 1.463; C2–C3, 1.345 (C2′–C3′, 1.345); Cu1···K1, 3.049 (Cu1′···K1′, 3.203); Cu1···K1′, 3.049 (Cu1′···K1, 3.204). (b) [{LiCuA′_2_}_2_]; Cu1–C1 (Cu1′–C1′), 1.985; Cu1–C10 (Cu1′–C10′), 1.973; C1–C2, 1.448; C1′–C2′, 1.445; C2–C3, (C2′–C3′), 1.364; C11–C12 (C11′–C12′), 1.366; Li1···C2, 3.432; Li1···C3, 3.112; Li1···C11′, 3.397; Li1···C12′ (Li1···C12), 3.092; Li1′···C11, 3.397; 3.092; Li1′···C2′, 3.432; Li′···C3′, 3.112; Li1–Cu1–Li1′, 121.32; Cu1–Li1′–Cu1′, 58.68. (c) [{LiCu­(C_3_H_5_)_2_}_2_]; Cu1–C10, 1.994; Cu1–C10′, 1.994; Cu1′–C1, 2.000; Cu1–C10′, 2.000; C1–C2, 1.460; C2–C3, 1.347; C1′–C2′, 1.460; C2′–C3′, 1.347; C10–C11, 1.457; C11–C12, 1.349; C10′–C11′, 1.457; C11′–C12′, 1.349; Li1···C1, 2.218; Li1···C2, 2.194; Li1···C3, 2.271; Li1···C10′, 2.389; Li1···C11′, 2.165; Li1···C12′, 2.276; Cu1···Li1, 2.479; Cu1···Li1′, 2.528; C1′–Cu1–C10, 1674.45; Li1–Cu1–Li1′, 104.72; Cu1–Li1–Cu1′, 73.82.

We next examined the case of the hypothetical dimer [{LiCuA′_2_}_2_], which is complex **1** with K^+^ replaced with Li^+^ (Figure 7b). During optimization without symmetry constraints, the dimeric structure remained intact and the dimensions within each CuA′_2_ unit are nearly unchanged from those in **1**; i.e., the average Cu–C distance is 1.98 Å, increasing by only 0.02 Å, and the C–Cu–C′ angle has decreased slightly (170.4°) from the value in **1** (175.6°). More importantly, the Li^+^ ions have moved away from the copper centers and can no longer be considered an integral part of the structure. Unlike in **1**, the Li···Cu distances (3.60, 3.83 Å), although within the sum of van der Waals radii (4.50 Å),^38b^ are far outside the sum of the covalent radii for the elements (2.60 Å).^34^

Contacts between the Li^+^ ions and the double-bonded carbons of the A′ ligands in [{LiCuA′_2_}_2_] (3.09–3.43 Å) are well beyond the distance for typical Li^+^–π interactions (about 2.25–2.35 Å).^37,63^ Distances between Li^+^ and methyl groups (Li1···C5′, C14) of 3.32 and 3.38 Å suggest that they are energetically marginal or negligible.^64^ It seems reasonable to consider the structure to be more ionic in nature than **1**, i.e., as [{Li^+^[CuA′_2_]^−^}_2_].

Finally, we studied the case of [LiCu­(C_3_H_5_)_2_] (Figure 7c). Once again, we began with the structure of **1**, replacing both the TMS groups with hydrogens and the K^+^ with Li^+^. Parts of the molecule remained relatively unchanged after optimization; the allyl ligands stay localized (the difference between single and double bonds is 0.11 Å, essentially the same as in **1**), and the average Cu–C bond length (1.997 Å) is slightly longer (by 0.04 Å) than in **1**. Without the constraints of the TMS ligands found in the calculation of [{LiCuA′_2_}_2_], the Cu–Li distances decreased by over 1 Å to 2.48–2.53 Å, which is well within the sum of the covalent radii for these elements (2.60 Å).^34^ The twist between the two CuA′_2_ units increased from 33.5°, as calculated for [{KCu­(C_3_H_5_)_2_}_2_], to nearly orthogonal (at 88.0°) compared to the nearly parallel CuA′_2_ units in **1** (3.6° twist angle). The (CuLi)_2_ ring remains significantly distorted, even more so than in **1**, with a 32.4° difference between Cu–Li–Cu′ and Li–Cu–Li′ angles, compared to 27.8° in the crystal. Because of this distortion, the copper atoms are much closer together (3.01 Å) than in any other structures (cf. 4.03 Å in **1**, and 4.26 Å in the optimized [{KCu­(C_3_H_5_)_2_}_2_]. Although this is outside the distance for a formal covalent Cu–Cu′ bond (2.64 Å),^34^ it is well below the sum of van der Waals radii (4.76 Å), and falls within the range associated with cuprophilic Cu­(I)···Cu­(I)′ interactions.^65^

## Conclusions

The potassium bis­(allyl)­cuprate [KCuA′_2_] has been identified as a rare, well-defined Gilman-like reagent that functions in neat hexanes, enabling 1,4-conjugate additions under conditions that reduce donor coordination. With the appropriate reagents, its synthesis can be accomplished under either mechanochemical or solvothermal conditions, as is the case for the gold analog [KAuA′_2_] and the neutral [{CuA′}_4_] and [{AuA′}_4_] tetramers.

A recurring theme throughout the synthetic experiments is the noninterchangeability of alkali cations. Lithium, sodium, and potassium ions do more than just balance charge, as they also affect structure, aggregation, and ion pairing. Along with the bulk of the A′ ligand, the potassium ion uniquely stabilizes the persistent σ-allyl Cu­(I)/Au­(I) -ate platforms. Conversely, switching to Li^+^ or Na^+^ changes the aggregation and leads to the formation of neutral allyl complexes. Calculations reveal that the metal cation–π interaction with the bulky allyl fragments is a key factor in stabilizing these structures, indicating that exploring other late-transition-metal organometallic compounds could profit from steric modifications to ligands, allowing access to additional homoleptic complexes instead of relying on auxiliary ligands (e.g., NHCs, coordinating ethers) to ensure kinetic stability.

## Supplementary Material





## Notes

The Supporting Information includes additional experimental, computational, and crystallographic details (PDF)
Optimized coordinates of structures (XYZ)
