# Supplementary material for: Sterically Stabilized Homoleptic Copper and Gold Allyl Complexes
Source: Organometallics. 2026 Feb 20;45(5):552–62. doi: 10.1021/acs.organomet.5c00460 (PMC12977057; doi:10.1021/acs.organomet.5c00460)
Supplement: Supplementary file 1 [file om5c00460_si_001.pdf]

SUPPLEMENTARY INFORMATION

for

Sterically Stabilized Homoleptic Copper and Gold Allyl Complexes

Dillon Button-Jennings,[a] Nathan D. Schley,[a] and Timothy P. Hanusa*[a]

[a]Department of Chemistry, Vanderbilt University, Nashville, Tennessee, 37235, USA.

Corresponding Author: t.hanusa@vanderbilt.edu

Experimental Procedures S2
Figure S1: ^{1}H NMR (400 MHz, C_6D_6) of [{KCuA´$_2$}$_2$] from Cu(I) S8
Figure S2: ^{13}C{^{1}H} NMR (100 MHz, C_6D_6) of [{KCuA´$_2$}$_2$] from Cu(I). S8
Figure S3: ^{1}H NMR (400 MHz, C_6D_6) of [{CuA´}$_4$] from Cu(II) S9
Figure S4: ^{1}H NMR (400 MHz, C_6D_6) of [{KAuA´$_2$}$_2$] from Au(I). S10
Figure S5: ^{13}C{^{1}H} NMR (100 MHz, C_6D_6) of [{KAuA´$_2$}$_2$] from Au(I) S10
Figure S6: : ^{1}H NMR (400 MHz, C_6D_6) of [{AuA´}$_4$] from Au(I). S11
Figure S7: ^{13}C{^{1}H} NMR (100 MHz, C_6D_6) of [{AuA´}$_4$] from Au(I). S11
Figure S8: ^{1}H NMR (400 MHz, $CDCl_3$) of (**5**) S12
Figure S9: ^{13}C{^{1}H} NMR (150 MHz, $CDCl_3$) of (**5**). S12
Figure S10: HSQC (150 MHz, CDCl) of (**5**) S13
Figure S11: ^{1}H NMR (400 MHz) of (**6**) in $CDCl_3$. S13
Figure S12: ^{13}C{^{1}H} NMR (150 MH, $CDCl_3$) of (**6**). S14
Figure S13: Interligand H···H´ contacts in [{CuA´}$_4$] that are ≤ 2.4 Å S15
Figure S14: [{CuA´}$_4$] and [{AuA´}$_4$] under UV radiation S16
Figure S15: Atom-In-Molecules (AIM) analysis for [{KCuA´$_2$}$_2$] and [{CuA´}$_4$] S17
Figure S16: Atom-In-Molecules (AIM) analysis for and [{KAuA´$_2$}$_2$] and [{AuA´}$_4$] S18
Table S1: Crystal Data and Summary of X-ray Data Collection S19
References S20

Experimental Procedures

General Considerations. All manipulations were performed with the rigorous exclusion of air and moisture using Schlenk or glovebox techniques. All glassware was dried by flame or in an oven at 140 °C overnight. Elemental analysis was performed at the University of Rochester CENTC Elemental Analysis Facility by Dr. William Brennessel. Proton and carbon ($^{13}C\{^{1}H\}$) spectra were obtained on an AV-400 spectrometer at 400 (^{1}H) and 100 (^{13}C) MHz. Proton and carbon spectra were referenced to the residual proton and ^{13}C resonances of C_6D_6 or $CDCl_3$. GC/MS data were collected on an Agilent Technologies 5977 GC/MSD.

Materials. Anhydrous metal halides ($CuCl_2$, $CuBr_2$, CuI_2, $AuCl_3$), [Cu(DMS)Br], 2-cyclohexenone, and methyl vinyl ketone were purchased from Sigma-Aldrich; [Au(DMS)Br] and [Au(py)Cl_3] were sourced from Strem Chemicals. The adducts [$CuCl_2$•$(PBu_3)_{0.5}$],[1] [$CuCl_2$•$(PPh_3)_{0.5}$],[1] and [$Cu(py)_2Cl_2$][2] were synthesized according to literature procedures. Li[A´] was prepared by the literature procedure,[3] and Na[A´] and K[A´] were prepared by transmetallation of Li[A´] with sodium or potassium *tert*-butoxide, respectively, in hexane solution. THF, Et_2O, toluene, and hexanes were purified over a MBRAUN solvent purification column. All solvents were stored in the glovebox over 4A molecular sieves. Deuterated benzene was purchased from Cambridge Isotopes, degassed and stored over 4A molecular sieves before use.

Mechanochemical protocol. Planetary milling was performed with a Retsch PM100 mill, a 50 mL zirconia grinding jar, and a safety clamp. Mechanochemical reactions were run by adding the solid reagents into a grinding jar with approximately 25 g of 5 mm zirconia ball bearings (0.34 g each).

Solid-state synthesis of [{KCuA´$_2$}$_2$] (1) from Cu(I). In a typical reaction, [Cu(DMS)Br] (0.077 g, 0.26 mmol) and K[A´] (0.120 g, 0.53 mmol) were added to a 100 mL zirconia Retsch milling jar with 25 g of zirconia ball bearings. The jar was clamped shut in a nitrogen glovebox and milled for 30 min at 600 rpm in a Retsch PM100 planetary mill. The jar was transferred back to the glovebox, and the light-brown solid was extracted with hexanes using a fine-porosity glass fritted funnel. The resulting yellow filtrate was dried under vacuum to yield **1** as a tan powder (0.135 g, 85%). ^{1}H NMR (600 MHz, C_6D_6) δ 0.262 (s, 36H, TMS), 3.16 (d, 4H), 7.04 (t, 2H). $^{13}C\{^{1}H\}$ NMR (150 MHz, C_6D_6) δ 0.21 (TMS), 70.6 ($C_{(1)}$,$C_{(3)}$), 162.1 ($C_{(2)}$). Calcd for $C_{18}H_{42}CuKSi_4$: C, 45.66, H, 8.94. Found: C, 47.04, H, 9.61. Although the values are somewhat high, the observed molar C/H ratio is 1:2.4, not far from the expected 1:2.3. The deviation is attributed to the thermal sensitivity of the compound; considerable care has to be used to prevent unwanted decomposition.

Solution synthesis of [{KCuA´$_2$}$_2$]. In a typical reaction, an oven-dried 20 mL glass vial is charged with [Cu(DMS)Br] (0.047 g, 0.23 mmol) and dissolved in ca. 5 mL of THF. To this colorless solution, K[A´] (0.100 g, 0.45 mmol) was added dropwise while stirring. The reaction darkened to a deep brown overnight. The solution was filtered through a Celite plug, and the filtrate was pulled to dryness. The crude brown solid was taken up with hexanes and filtered through an oven-dried Celite plug to afford a golden-brown filtrate.

The filtrate was dried under vacuum to yield a tan powder (0.067 g, 64%) identified as **1** from its ^{1}H NMR spectrum.

Solid-state synthesis of [{KCuA´$_2$}$_2$] (1) from Cu(II). In a typical reaction, [Cu(py)$_2$Cl$_2$][2] (0.049 g, 0.17 mmol) and K[A´] (0.076 g, 0.34 mmol) were added to a 100 mL zirconia Retsch milling jar with 25 g of zirconia ball bearings. The jar was clamped shut in a nitrogen glovebox and milled for 30 min at 600 rpm in a Retsch PM100 planetary mill. The jar was returned to the glovebox, and the turquoise solid was extracted with hexanes through a fine-porosity glass fritted funnel. The resulting orange filtrate was dried under vacuum to yield a transparent, brown oil (0.058 g) containing 61% of [KCuA´$_2$]$_2$ determined with NMR (net 0.035 g, 36%). The remaining 39% of the crude product is a mixture of the propene and the coupled dimer (1,3,4,6-tetrakis(trimethylsilyl)hexa-1,5-diene). The crude oil crystallizes overnight to leave colorless crystals identified as **3** through single crystal X-ray diffraction. ^{1}H NMR (400 MHz, C_6D_6) δ 0.27 (s, 18H, TMS), 3.21 (d, 2H, terminal CH), 7.07 (t, 1H, center CH).

Attempted solution synthesis of [{KCuA´$_2$}$_2$] (1) from Cu(II). In a typical reaction, an oven-dried 20 mL glass vial was charged with [Cu(py)$_2$Cl$_2$] (0.043 g, 0.27 mmol) and dissolved in ca. 5 mL of toluene. To this turquoise solution, K[A´] (0.121 g, 0.54 mmol) was added dropwise while stirring. The appearance of the reaction darkened to a deep yellow-brown upon addition and then blackened overnight. The solvent was removed under vacuum, and the black solid was extracted with hexanes and filtered through an oven-dried celite plug to afford a clear filtrate. The filtrate was dried under vacuum to yield an off-white crystalline solid, identified as the coupled dimer (1,3,4,6-tetrakis(trimethylsilyl)hexa-1,5-diene), based on its characteristic NMR spectrum.[4]

Attempted solution synthesis of [{KCuA´$_2$}$_2$] (1) from Cu(II). In a typical reaction, an oven-dried 20 mL glass vial was charged with [Cu(py)$_2$Cl$_2$] (0.043 g, 0.27 mmol) and dissolved in ca. 5 mL of toluene. To this turquoise solution, K[A´] (0.121 g, 0.54 mmol) was added dropwise while stirring. The reaction mixture darkened to a deep yellow brown upon addition and then blackened overnight. The solvent was removed under vacuum, and the black solid was extracted with hexanes and filtered through an oven-dried celite plug to afford a clear filtrate. The filtrate was dried under vacuum to yield an off-white, crystalline solid, identified as the coupled dimer (1,3,4,6-tetrakis(trimethylsilyl)hexa-1,5-diene), based on its characteristic NMR spectrum.[4]

Solid-state synthesis of [{CuA´}$_4$] (2). In a typical reaction, [Cu(py)$_2$Cl$_2$] (0.043 g, 0.15 mmol) and Na[A´] (0.078 g, 0.37 mmol) were added to a 100 mL zirconia Retsch milling jar with 25 g of zirconia ball bearings. The jar was clamped shut in an ether-free nitrogen glovebox and milled for 15 min at 600 rpm in a Retsch PM100 planetary mill. The jar was transferred back to the ether-free glovebox, and the purple solid was extracted with hexanes using a fine-porosity glass fritted funnel. The resulting yellow filtrate was dried under vacuum to expose an oil containing yellow blocks (0.035 g, 17%). The yellow blocks were identified as **2** with SCXRD; however, the crude oil contained equal amounts of the coupled dimer (1,3,4,6-tetrakis(trimethylsilyl)hexa-1,5-diene), as determined from ^{1}H NMR spectroscopy. Small amounts of light-colored crystals could be obtained by cooling a hexanes solution to -40 ·C; these were adequate for single crystal X-ray diffraction ^{1}H NMR

(400 MHz, C_6D_6) δ 0.28 (s, 18H, TMS), 3.42 (d, 2H, terminal CH), 6.26 (t, 1H, center CH). The compound was too thermally unstable to obtain elemental analysis.

Attempted solution synthesis of [{CuA´}$_4$] (2) from Cu(II). In a typical reaction, an oven-dried 20 mL glass vial was charged with [Cu(py)$_2$Cl$_2$] (0.043 g, 0.15 mmol) and dissolved in ca. 5 mL of toluene. To this turquoise solution, Na[A´] (0.078 g, 0.37 mmol) was added dropwise while stirring. The reaction darkens to a deep yellow-brown upon addition and then blackens overnight. The solvent was removed under vacuum, and the black solid was extracted with hexanes and filtered through an oven-dried Celite plug to afford a clear filtrate. The filtrate was dried under vacuum to yield an off-white crystalline solid. This solid was identified as the coupled dimer (1,3,4,6-tetrakis(trimethylsilyl)hexa-1,5-diene) from its characteristic ^{1}H NMR spectrum.[4]

Attempted solid-state synthesis of [{CuA´}$_4$] (2) from Cu(I) using Na[A´]. In a typical reaction, [Cu(DMS)Br] (0.035 g, 0.17 mmol) and Na[A´] (0.035 g, 0.16 mmol) were added to a 100 mL zirconia Retsch milling jar with 25 g of zirconia ball bearings. The jar was clamped shut in a nitrogen glovebox and milled for 30 min at 600 rpm in a Retsch PM100 planetary mill. The jar was returned to the glovebox, and the purple solid was extracted with hexanes using a fine-porosity glass-fritted funnel. The resulting colorless filtrate was dried under vacuum to leave a colorless solid identified as the coupled dimer (1,3,4,6-tetrakis(trimethylsilyl)hexa-1,5-diene) from its characteristic ^{1}H NMR spectrum.[4]

Attempted solid-state synthesis of [{CuA´}$_4$] (2) from Cu(I) using K[A´]. In a typical reaction, [Cu(DMS)Br] (0.122 g, 0.59 mmol) and K[A´] (0.131 g, 0.58 mmol) were added to a 100 mL zirconia Retsch milling jar with 25 g of zirconia ball bearings. The jar was clamped shut in a nitrogen glovebox and milled for 30 min at 600 rpm in a Retsch PM100 planetary mill. The jar was returned to the glovebox, and the purple solid was extracted with hexanes using a fine-porosity frit. The resulting light-yellow filtrate was dried under vacuum, yielding a tan powder identified from its ^{1}H NMR spectrum as the cuprate **1**.

Solid-state synthesis of [{KAuA´$_2$}$_2$] (3) from Au(I). In a typical reaction, [Au(DMS)Cl] (0.0427 g, 0.14 mmol) and K[A´] (0.067 g, 0.29 mmol) were added to a 100 mL zirconia Retsch milling jar with 25 g of zirconia ball bearings. The jar was clamped shut in a nitrogen glovebox and milled for 30 min at 600 rpm in a Retsch PM100 planetary mill. The jar was transferred back to the glovebox and the purple solid was extracted with hexanes through a fine-porosity glass fritted funnel. The resulting pale-yellow filtrate was dried under vacuum to leave a yellow crystalline solid comprising **3** (0.060 g, 29%) with trace amounts of 1,3,4,6-tetrakis(trimethylsilyl)hexa-1,5-diene and **4**. ^{1}H NMR (400 MHz, C_6D_6): δ 0.23-0.44 (m, 36H, TMS), 2.05 (d, 1H, Au-CH), 2.18 (d, 1H, Au-CH), 4.04 (d, 1H, HC=C), 4.14 (d, 1H, beta-CH), 6.85 (m, 2H, alpha-CH). ^{13}C{^{1}H} NMR (100 MHz, C_6D_6) δ 0.1 (TMS), 1.7 (TMS), 47.1 ($C_{(1)}$), 67.6 ($C_{(2)}$), 162.6 ($C_{(3)}$). Anal. Calcd for $C_{18}H_{42}AuKSi_4$: C, 35.62; H, 6.97. Found: C, 35.68; H, 7.13 (average of two determinations).

Solid-state synthesis of [{KAuA´$_2$}$_2$] (3) from Au(III). In a typical reaction, [Au(py)Cl$_3$] (0.059 g, 0.14 mmol) and K[A´] (0.100 g, 0.42 mmol) were added to a 100 mL zirconia Retsch milling jar with 25 g of zirconia ball bearings. The jar was clamped shut in a nitrogen glovebox and milled for 30 min at 600 rpm in a Retsch PM100 planetary mill. The jar

was returned to the glovebox, and the purple solid was extracted with hexanes using a fine-porosity glass fritted funnel. The resulting red filtrate was dried under vacuum to yield a transparent, red-tinged oil. The oil slowly crystallizes over days, exposing colorless rhomboids of **3** (0.0473 g, 50%). The aurate can be further purified by extracting the crude hexane filtrate with acetonitrile.

Solution synthesis of [{KAuA´$_2$}$_2$] (3) from Au(III). In a typical reaction, an oven-dried 20 mL glass vial is charged with [Au(py)Cl$_3$] (0.043 g, 0.11 mmol) and dissolved in ca. 5 mL of toluene. To this yellow solution, K[A´] (0.075 g, 0.33 mmol) was added dropwise while stirring. The reaction darkens to a deep purple overnight. The solution was transferred to an Erlenmeyer flask, and the solvent was removed under vacuum. The crude purple solid was taken up with hexanes and filtered through an oven-dried Celite plug, affording a red filtrate. The red filtrate was dried under vacuum to yield a transparent, red-tinged oil that crystallized over the course of days to form colorless rhomboids, identified as **3** (trace yield).

Solid-state synthesis of [{AuA´}$_4$] (4) from Au(I). In a typical reaction, [Au(DMS)Cl] (0.037 g, 0.13 mmol) and K[A´] (0.029 g, 0.13 mmol) were added to a 100 mL zirconia Retsch milling jar with 25 g of zirconia ball bearings. The jar was clamped shut in a nitrogen glovebox and milled for 30 min at 600 rpm in a Retsch PM100 planetary mill. The jar was transferred back to the glovebox, and the purple solid was extracted with hexanes through a fine-porosity glass fritted funnel. The resulting light brown filtrate was dried under vacuum to leave a yellow crystalline solid comprising **4** (0.019 g, 40%) with trace amounts of 1,3,4,6-tetrakis(trimethylsilyl)hexa-1,5-diene. ^{1}H NMR (400 MHz, C_6D_6): δ 0.28 (s, 18H, TMS), 4.01 (d, 2H, terminal proton), 6.25 (t, 1H, central proton).^{13}C{^{1}H} NMR (100 MHz, C_6D_6) δ 0.45 (TMS), 65.9 ($C_{(1),(3)}$), 149.5 ($C_{(2)}$). Anal. Calcd for $C_{36}H_{82}Au_4Si_8$: C, 28.31; H, 5.41. Found: C, 28.18; H, 5.36.

Solid-state synthesis of [{AuA´}$_4$] (4) from Au(III). In a typical reaction, $AuCl_3$ (0.05 g, 0.16 mmol) and K[A´] (0.097 g, 0.43 mmol) were added to a 100 mL zirconia Retsch milling jar with 25 g of zirconia ball bearings. The jar was clamped shut in a nitrogen glovebox and milled for 30 min at 600 rpm in a Retsch PM100 planetary mill. The jar was transferred back to the glovebox and the purple solid was extracted with hexanes through a fine porosity glass fritted funnel. The resulting red filtrate was dried under vacuum to yield a transparent, red-tinged oil. The oil crystallizes overnight to leave pale-yellow rhomboids in trace yield (<10%). The NMR indicates that the propene (1,3-$SiMe_3$)$_2C_3H_4$ and (1,3,4,6-tetrakis(trimethylsilyl)hexa-1,5-diene) are the side products by comparing them to reported NMR values.[1]

Attempted solution synthesis of [{AuA´}$_4$] (4) from Au(III). In a typical reaction, an oven-dried 20 mL glass vial was charged with $AuCl_3$ (0.043 g, 0.14 mmol) and dissolved in ca. 5 mL of toluene. To this red solution, K[A´] (0.097 g, 0.43 mmol) was added dropwise while stirring. The reaction darkened to a deep brown overnight. The solution was transferred to an Erlenmeyer flask, and the solvent was removed under vacuum. The purple solid was extracted with hexanes and filtered through an oven-dried Celite plug to afford light brown filtrate. The filtrate was dried under vacuum to yield a white crystalline

solid. This solid was identified as the coupled dimer (1,3,4,6-tetrakis(trimethylsilyl)hexa-1,5-diene), identified from its characteristic NMR spectrum.[4]

3-(1,3-bis(trimethylsilyl)allyl)cyclohexan-1-one (5). In a typical reaction, an oven-dried 20 mL glass vial was charged with **3** (0.378 g, 0.79 mmol) and dissolved in ca. 10 mL of hexanes. To this pale-yellow solution, 2-cyclohexanone (0.07 mL, 0.73 mmol) was added dropwise while stirring at -78 °C. After 30 min, the orange solution was quenched via dropwise addition of an aqueous solution of saturated ammonium chloride and allowed to warm to room temperature over the next 30 min. The crude organic layer was extracted with Et_2O, dried over $MgSO_4$, filtered, and the solvent was then evacuated under vacuum. The crude residue was purified via flash chromatography (SiO_2, 12.5% Et_2O in hexanes) to afford **5** (0.067 g, 33% yield) alongside residual starting material (7:1). ^1H NMR (400 MHz, $CDCl_3$): δ 0.02 (s, 9H, TMS), 0.04 (s, 9H, TMS), 2.07 (m, 5H), 2.3 (mm, 5H), 2.72 (t, 1H), 5.47 (d, 1H), 5.9 (dd, 1H). $^{13}C\{^1H\}$ NMR (150 MHz, $CDCl_3$) δ -0.23 (TMS), 0.59 (TMS), 27.28, 31.3, 41.3, 42.9, 46.9, 50.7, 132.8, 146.2, 214.1. GC/MS: calculated for $[C_{15}H_{30}OSi_2]^+$ (M^+) 282.18; found 282.1.

5,7-bis(trimethylsilyl)hept-6-en-2-one (6). In a typical reaction, an oven-dried 20 mL glass vial was charged with **3** (0.361 g, 0.76 mmol) and dissolved in ~10 mL of hexanes. To this pale-yellow solution, methyl vinyl ketone (0.06 mL, 0.72 mmol) was added dropwise while stirring at -78 °C. After 30 min, the deep orange solution is quenched via dropwise addition of an aqueous solution of saturated ammonium chloride and allowed to warm to room temperature over the next 30 min. The crude organic layer was extracted with Et_2O, dried over $MgSO_4$, filtered, and the solvent was then evacuated under vacuum. The crude residue was purified via flash chromatography (SiO_2, 12.5% Et_2O in hexanes) to afford **6** (0.045 g, 34% yield) as a colorless oil. ^1H NMR (400 MHz, $CDCl_3$): δ -0.03 (s, 9H, TMS), 0.03 (s, 9H, TMS), 1.5 (m, 1H), 1.6 (m, 1H), 1.7 (m, 1H), 2.1 (s, 3H, $-CH_3$), 2.34 (m, 1H), 2.49 (m, 1H), 5.4 (d, 1H, HC=CH), 5.77 (dd, 1H, HC=CH). $^{13}C\{^1H\}$ NMR (150 MHz, $CDCl_3$) δ -3.2 (TMS), 0.82 (TMS), 22.42 (CH3), 30.32, 37.96, 43.66, 128 (C=C), 147 (C=C), 209.71 (C=O). GC/MS: calculated for $[C_{13}H_{28}OSi_2]^+$ (M^+) 256.17; found 256.1.

X-ray Crystallography. X-ray crystallographic data were collected on a Rigaku Oxford Diffraction Supernova diffractometer. Crystal samples were handled under immersion oil and quickly transferred to a cold nitrogen stream. The crystals were kept at 100 K during data collection. Under Olex2,[5] the structures were solved with the SHELXT[6] structure solution program using intrinsic phasing and refined with the SHELXL[7] refinement package using least-squares minimization. All non-hydrogen atoms were refined with anisotropic displacement parameters. Specific details for the hydrogen atoms in each structure are as follows:

[{KCuA´$_2$}$_2$] (1) Allyl hydrogens were located in the difference map and refined without restraint. The methyl group hydrogens were modeled in idealized positions and refined as a rotating group.

[{CuA´}$_4$] (2) Allyl hydrogens were located in the difference map and refined without restraint. The methyl group hydrogens were modeled in idealized positions and refined as a rotating group.

[{KAuA´$_2$}$_2$] (3) Allyl hydrogens were located in the difference map and refined without restraint. The methyl group hydrogens were modeled in idealized positions and refined as a rotating group.

[{AuA´}$_4$] (4) Disorder in gold atom positions was modeled over two positions with similarity restraints placed on the atomic thermal parameters. Allyl and methyl group hydrogen atoms were added at idealized locations.

General Procedures for Calculations. Geometry optimization and frequency calculations were performed with the Gaussian 16 (Linux and Windows) suite of programs.[8] The B3PW91 functional, which incorporates Becke's three-parameter exchange functional with the 1991 gradient-corrected correlation functional of Perdew and Wang, was used for most calculations.[9] To supply dispersion corrections, Grimme's D3 correction[10] with additional Becke-Johnson damping was used.[11] Unless otherwise noted, the def2-TZVP basis set was used on all atoms.[12] Frequency calculations were also performed at the triple-zeta level. Multiwfn (3.8dev) was used for AIM calculations and bond order estimations.[13]

Figure S1: ^{1}H NMR (400 MHz, C_6D_6) of [{KCuA´$_2$}$_2$] from Cu(I).

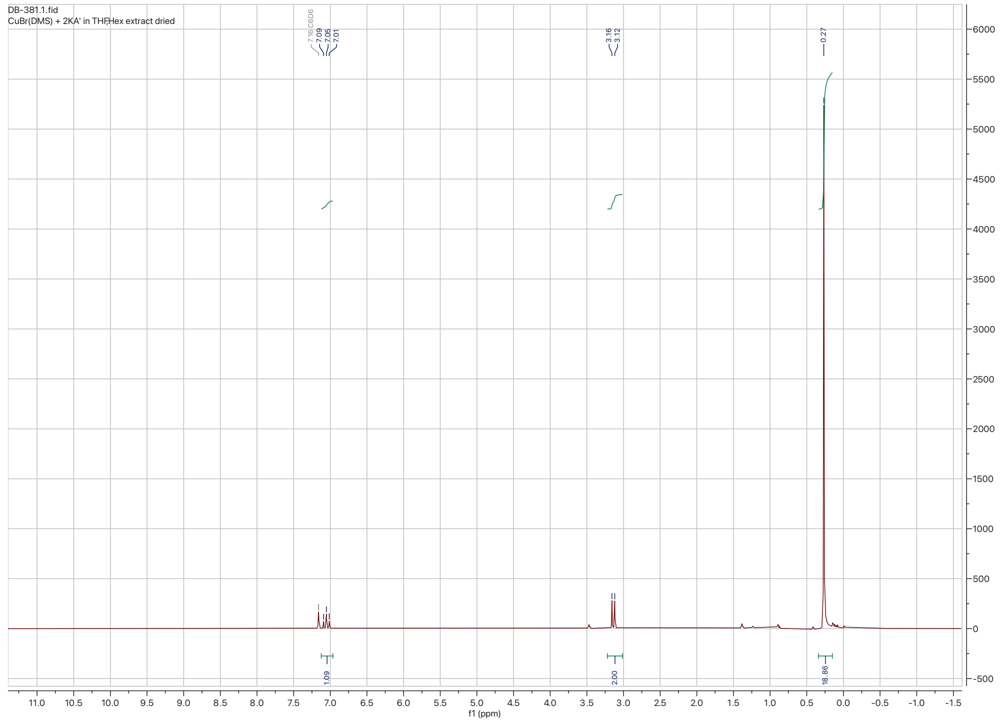

Figure S2: ^{13}C{^{1}H} NMR (100 MHz, C_6D_6) of [{KCuA´$_2$}$_2$] from Cu(I).

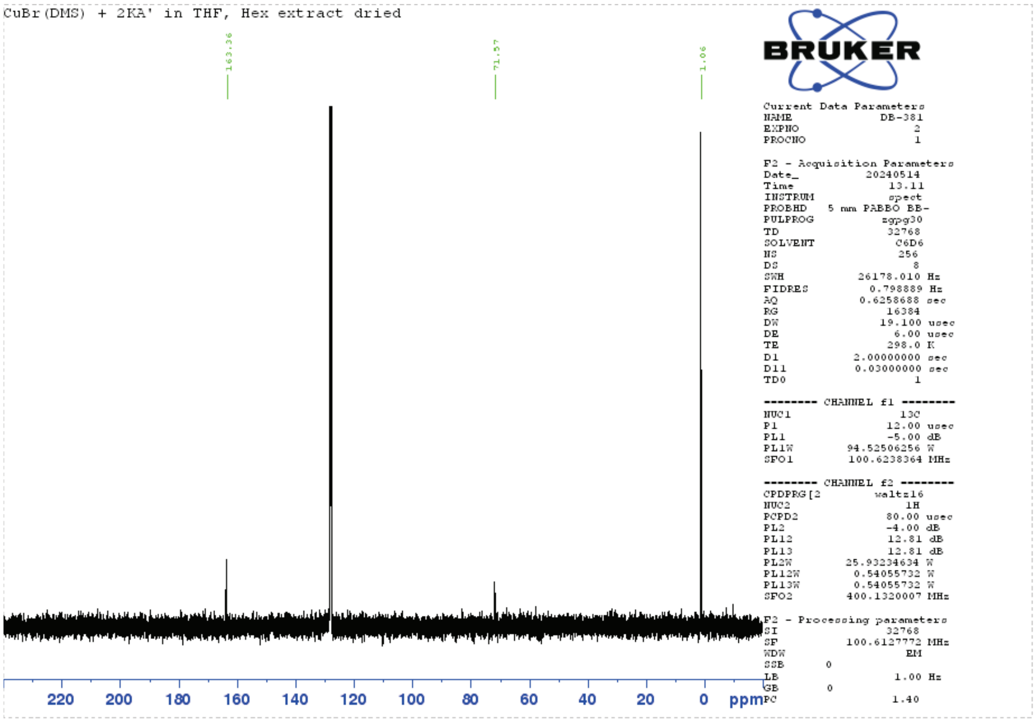

Figure S3: a) ^{1}H NMR (400 MHz, C_6D_6) of [{CuA´}$_4$] from Cu(II); b) Enlargement of the region from ca. δ 3.5–6.4.

a)

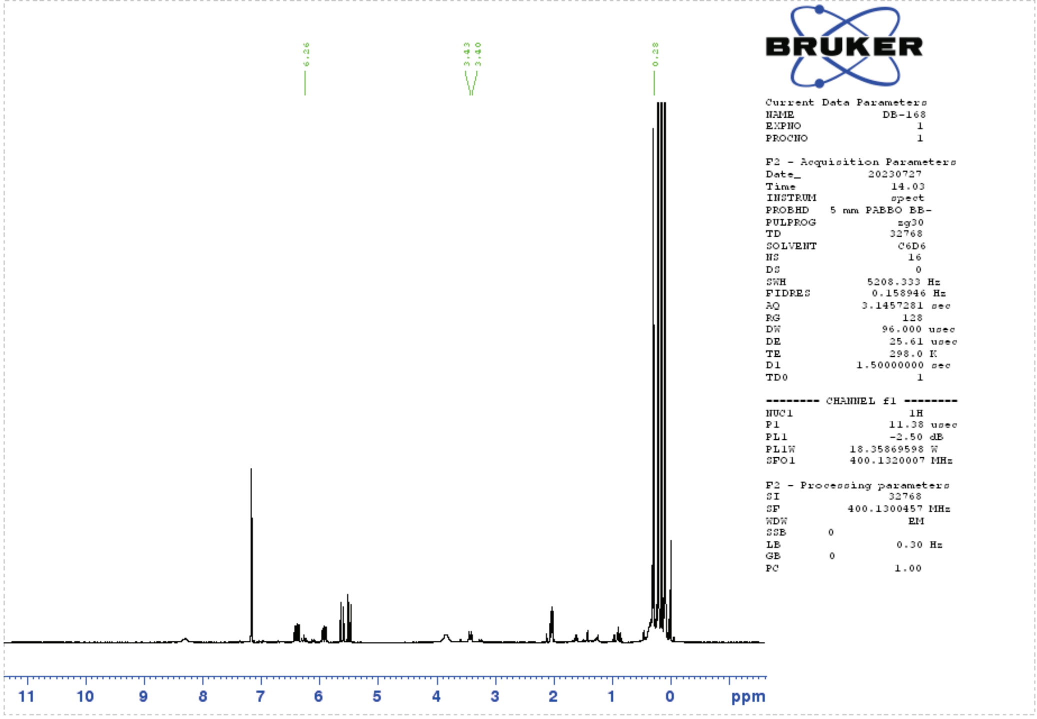

b)

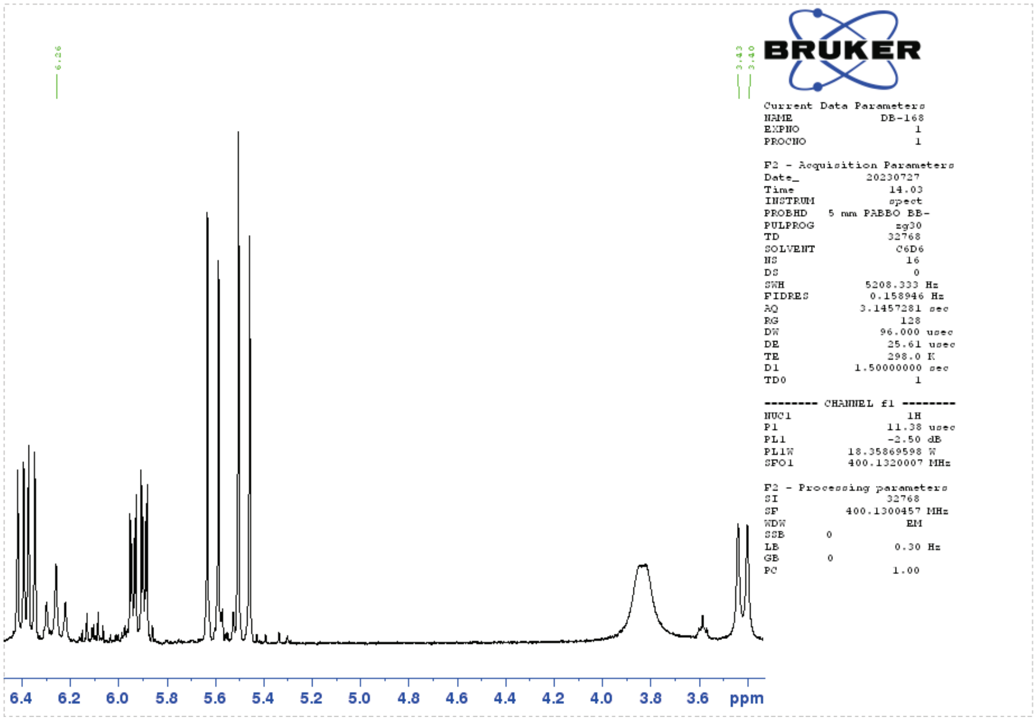

Figure S4: ^{1}H NMR (400 MHz, C_6D_6) of [{KAuA´$_2$}$_2$] from Au(I).

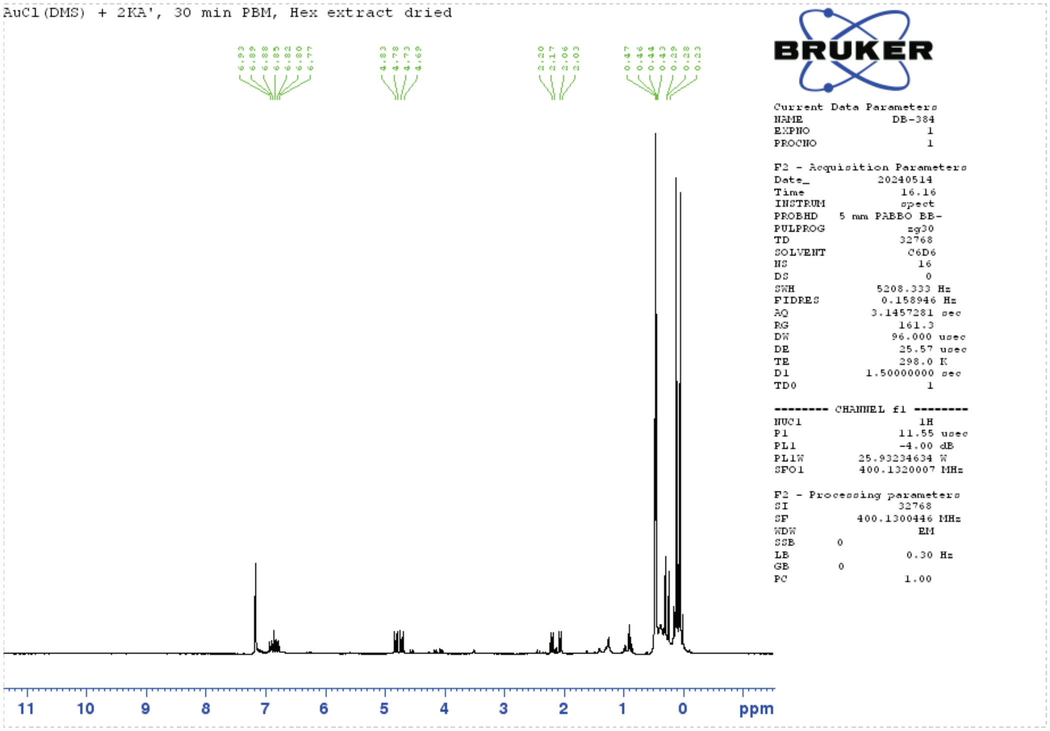

Figure S5: $^{13}C\{^{1}H\}$ NMR (100 MHz, C_6D_6) of [{KAuA´$_2$}$_2$] from Au(I).

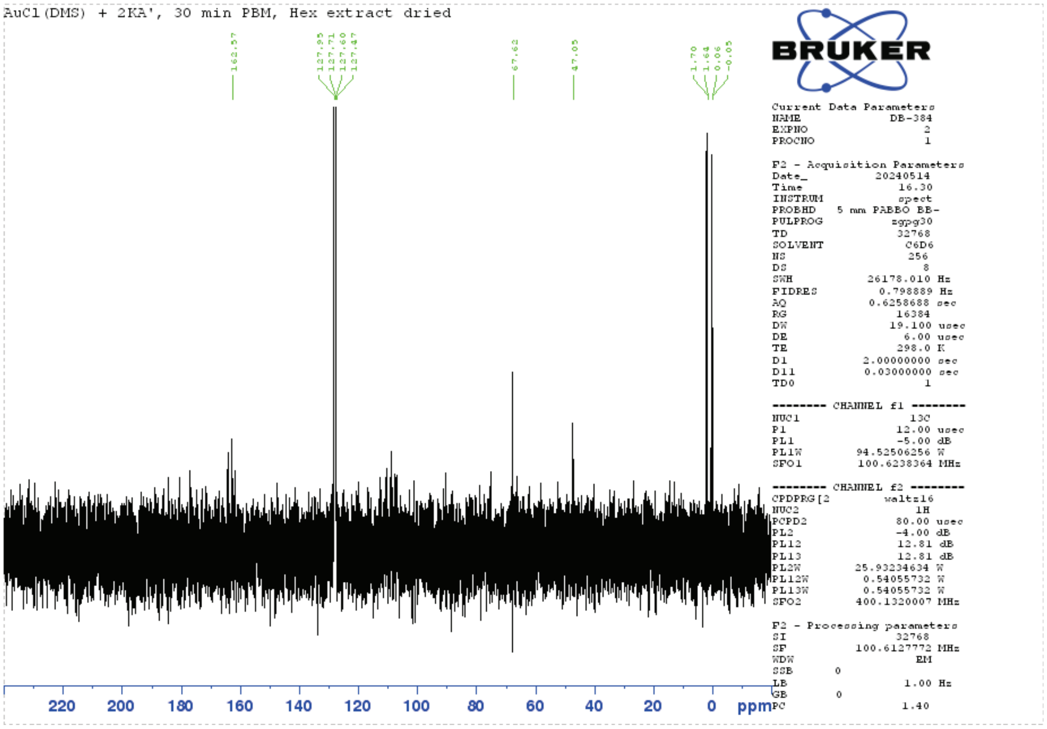

Figure S6: ^{1}H NMR (400 MHz, C_6D_6) of [{AuA´}$_4$] from Au(I).

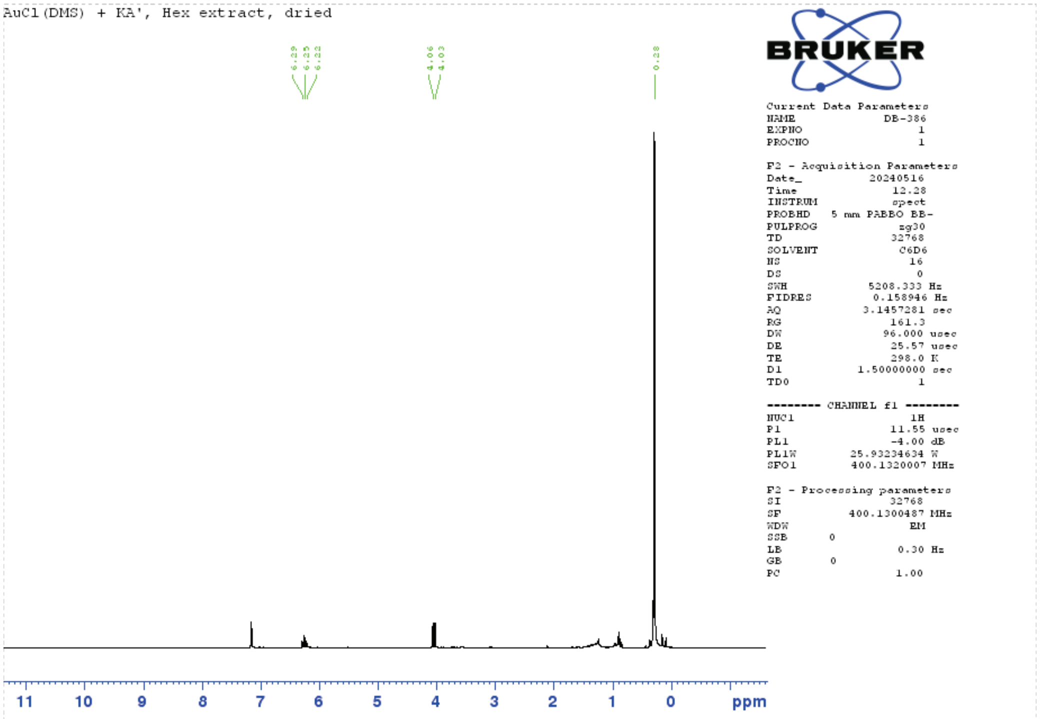

Figure S7: ^{13}C{^{1}H} NMR (100 MHz, C_6D_6) of [{AuA´}$_4$] from Au(I).

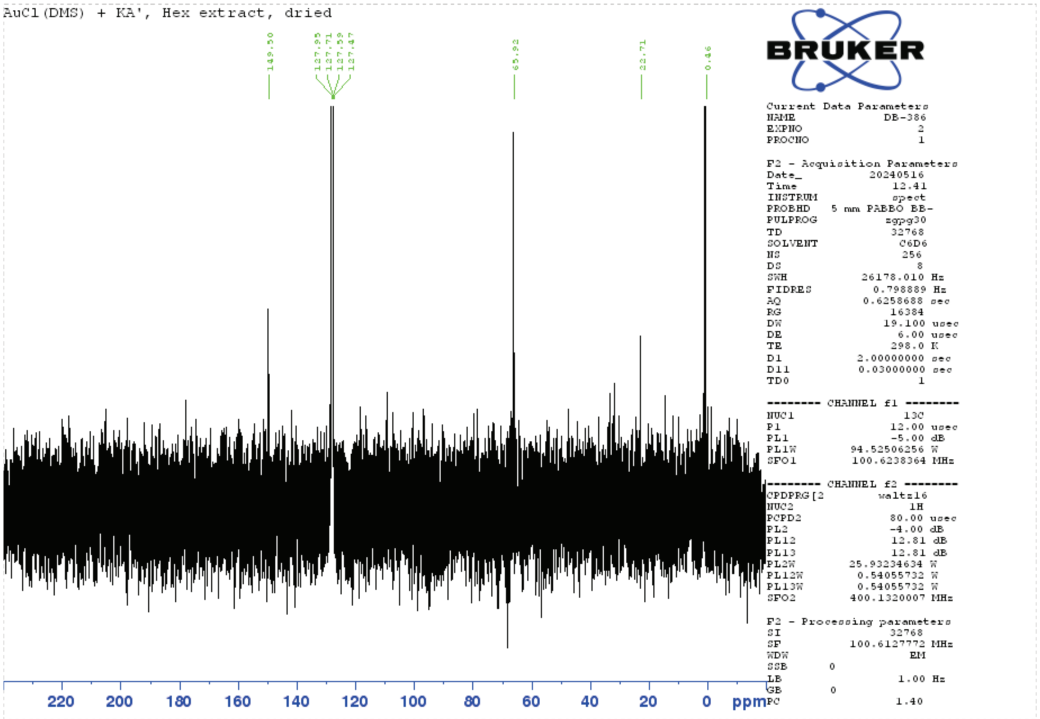

Figure S8: ^{1}H NMR (400 MHz, $CDCl_3$) of (**5**).

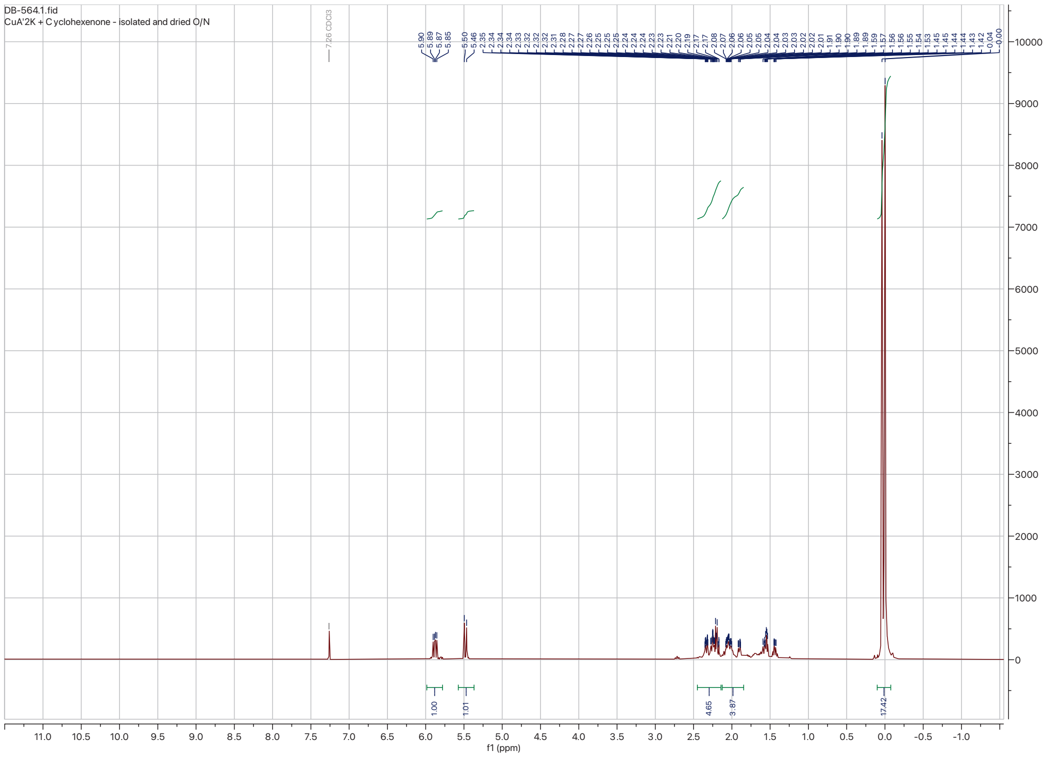

Figure S9: ^{13}C{^{1}H} NMR (150 MHz, $CDCl_3$) of (**5**).

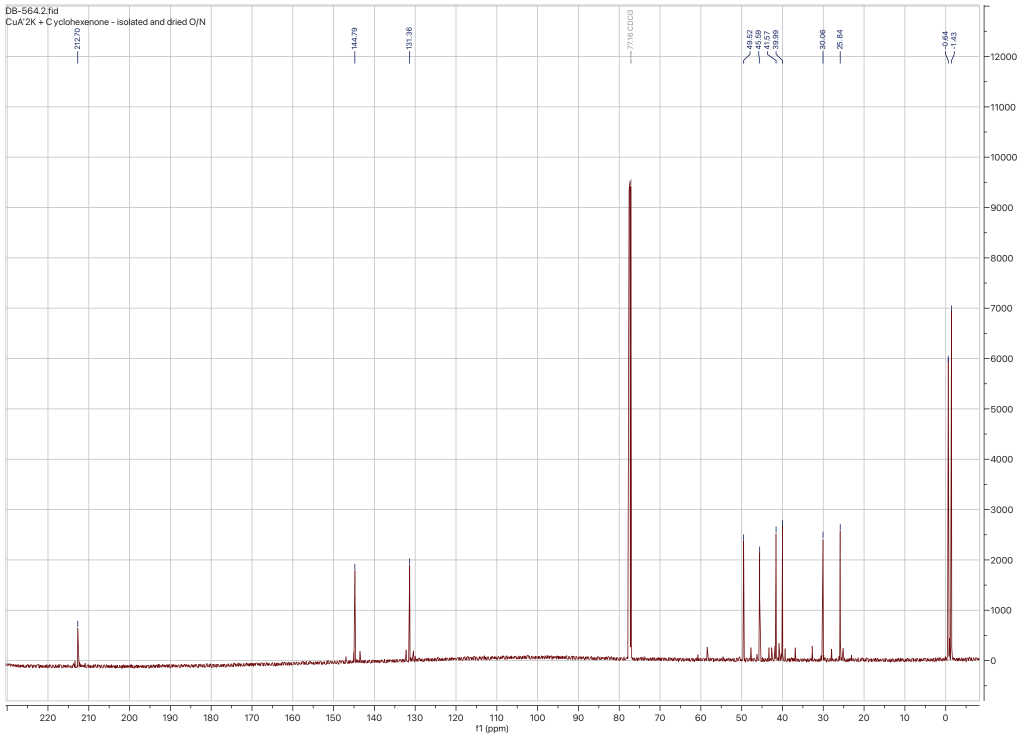

Figure S10: HSQC (150 MHz, CDCl) of (**5**).

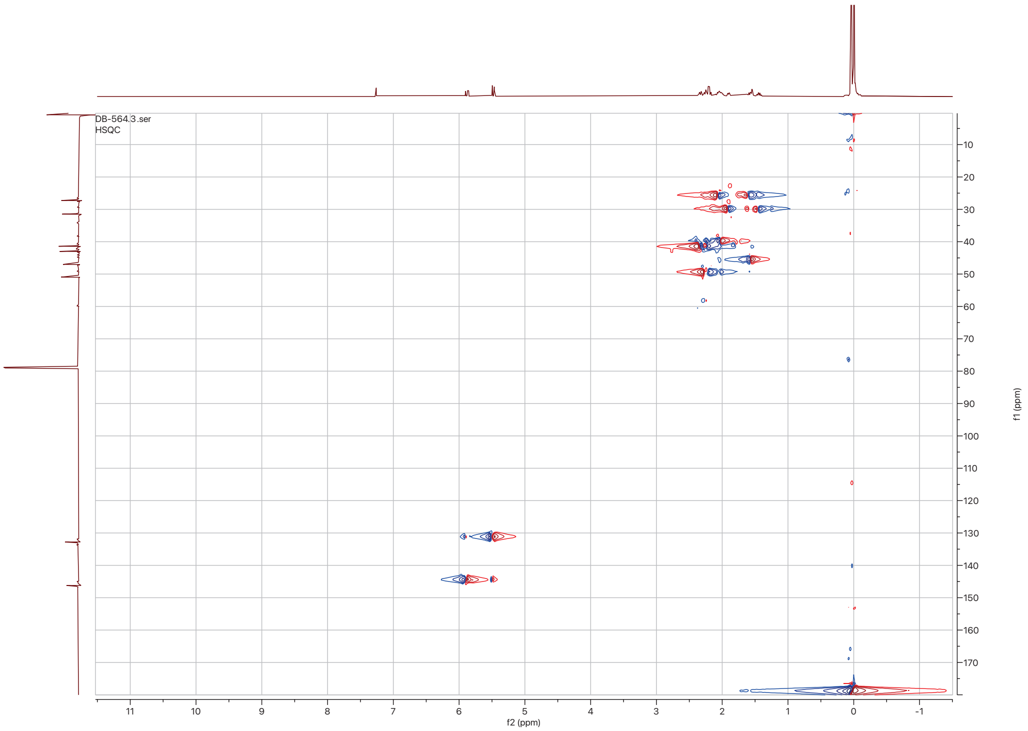

Figure S11: ^{1}H NMR (400 MHz) of (**6**) in $CDCl_3$.

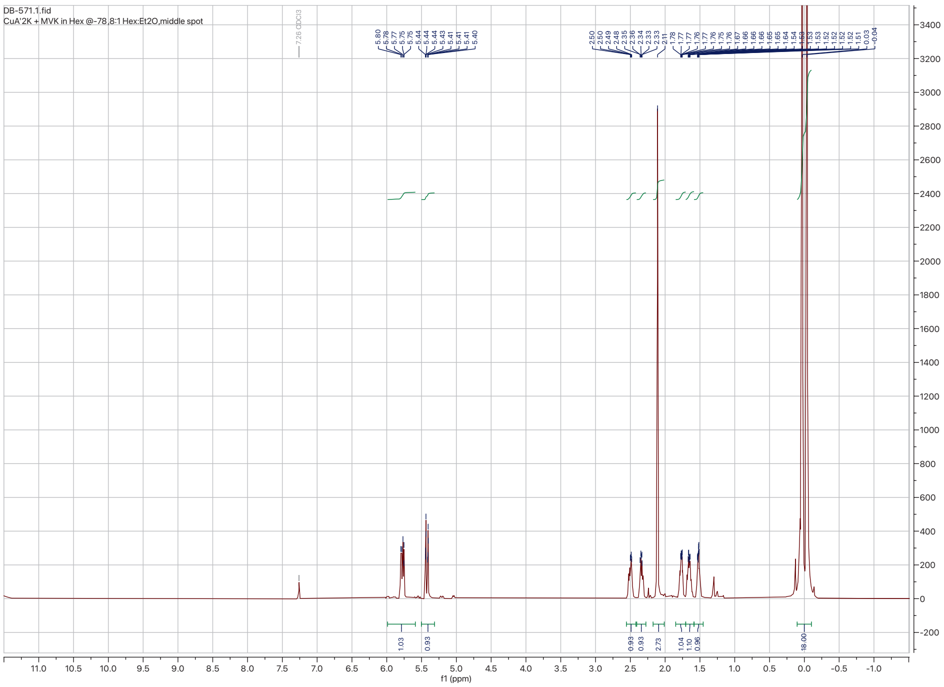

Figure S12: $^{13}C\{^{1}H\}$ NMR (150 MH, $CDCl_3$) of (**6**).

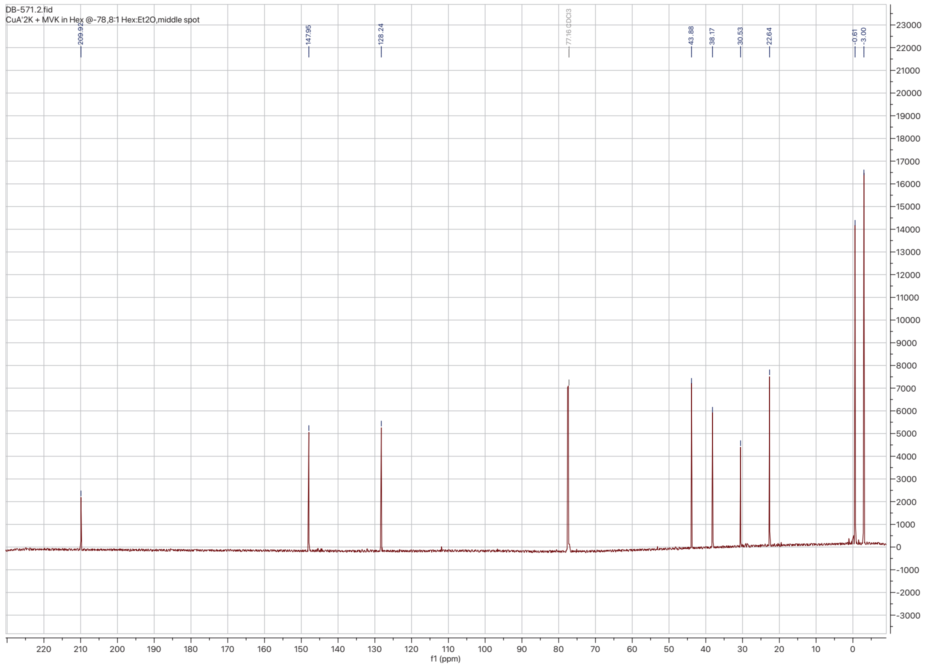

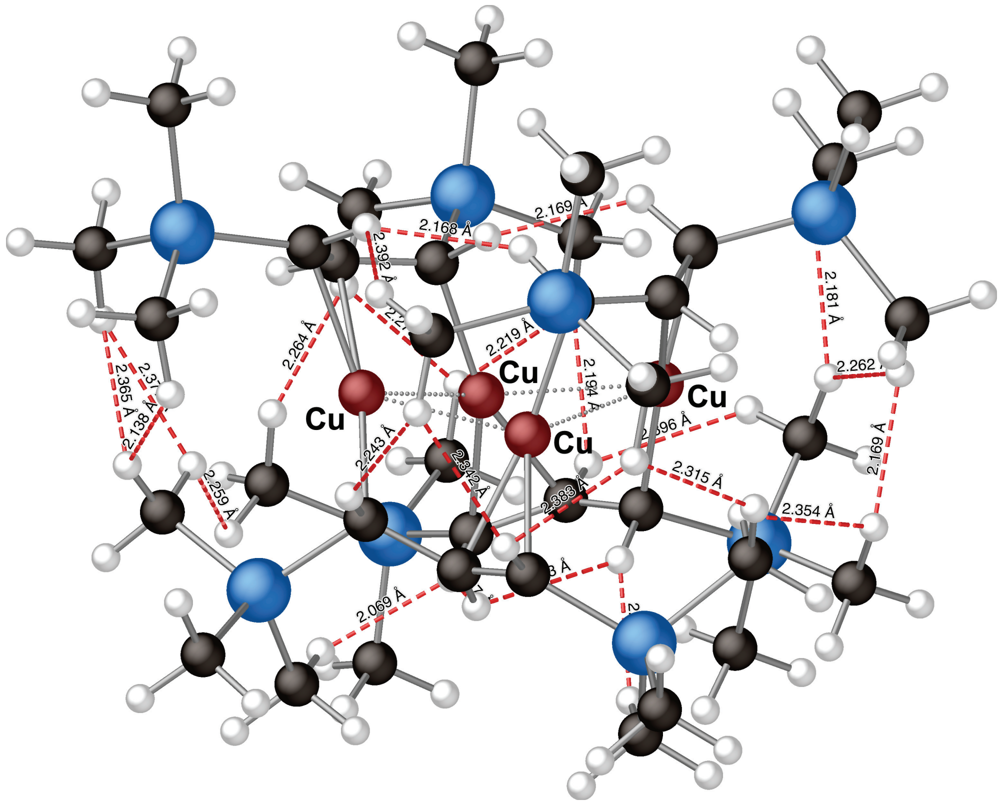

Figure S13. Interligand H···H´contacts in [{CuA´}$_4$] that are ≤ 2.4 Å (dashed red lines). These serve as proxies for the strength of dispersion forces. Distances calculated based on DFT-optimized structure (MN15L/def2tzvp(C,Si); def2svp(C,H)).

Figure S14. a) $[\{CuA´\}_4]$ under 254 nm radiation. b) $[\{AuA´\}_4]$ under 365 nm radiation.

a) 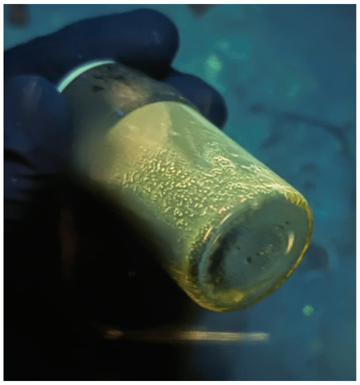

b) 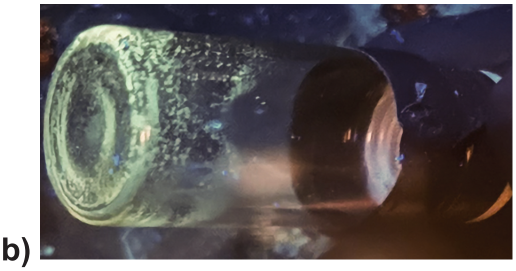

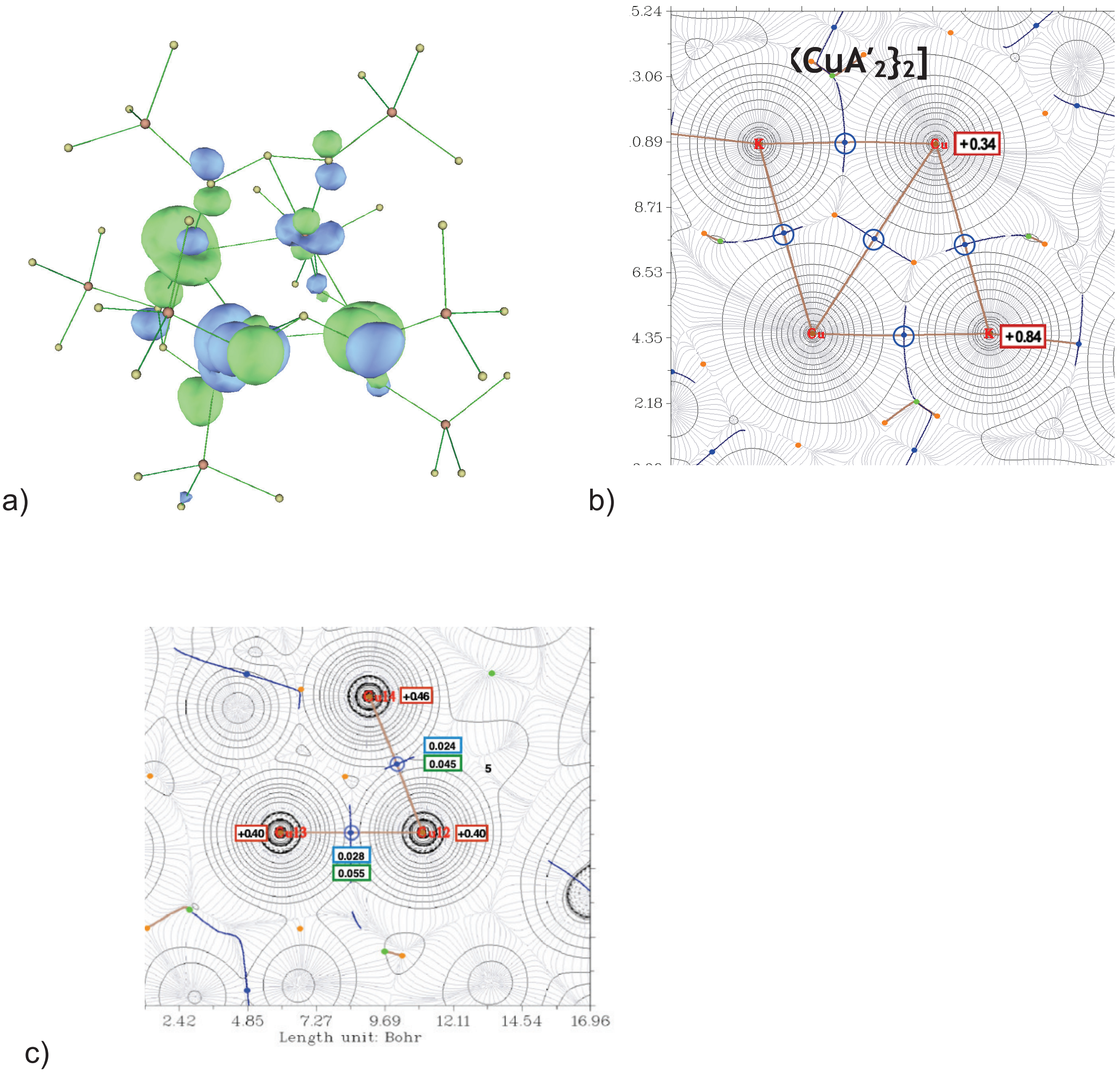

a) b) c)

Figure S15 a) HOMO of [{CuA´}$_4$] (**2**) and Atom-In-Molecules (AIM) analysis (MN15L/def2tzvp(M,Si); def2svp(C,H)) showing bond paths and bond-critical-points (circled) for (b) [{KCuA´$_2$}$_2$] (**1**) and (c) [{CuA´}$_4$] (**2**).The electron density, ρ(r) (blue box), and Laplacian, $\nabla^2\rho(r)$ (green box), in the bcp's are given in atomic units. Red boxes show AIM charges for the metal atoms.

The HOMO-LUMO gap in **2** is 3.85 eV (88.7 kcal mol^{-1}). Owing to the somewhat irregular shape of the Cu$_4$ unit, the Cu···Cu´ Mayer bond orders vary between 0.15 and 0.35 (ave = 0.28), slightly higher than in **4**.

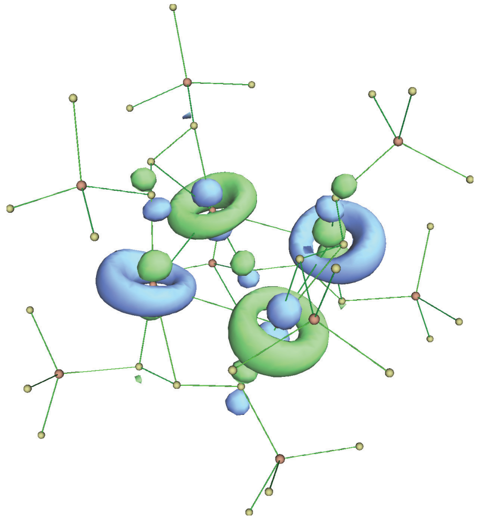

a)

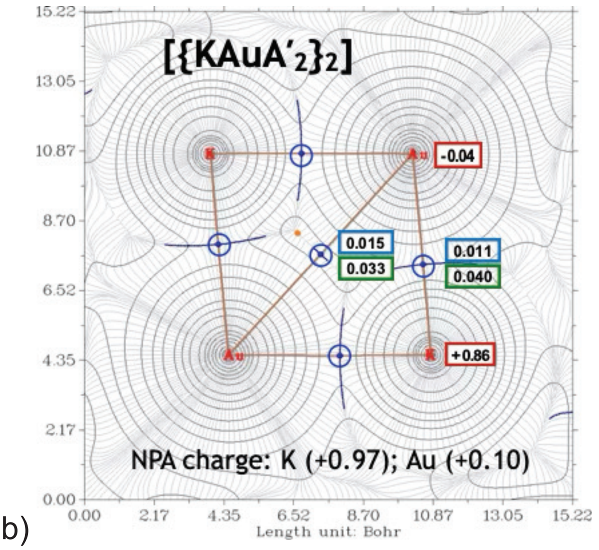

b)

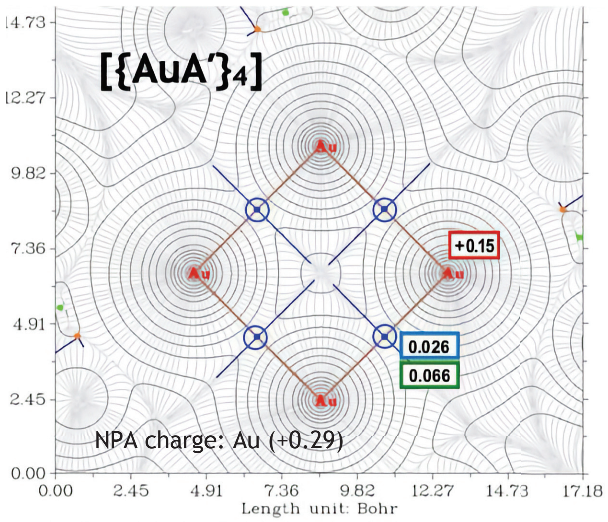

c)

Figure S16. a) HOMO of [{AuA´}$_4$] (**2**) and Atom-In-Molecules (AIM) analysis (MN15L/def2tzvp(M,Si); def2svp(C,H)) showing bond paths and bond-critical-points (circled) for (b) [{KAuA´$_2$}$_2$] (**3**) and (c) [{AuA´}$_4$] (**4**).The electron density, ρ(r) (blue box), and Laplacian, $\nabla^2\rho(r)$ (green box), in the bcp's are given in atomic units. Red boxes show AIM charges for the metal atoms.

The HOMO-LUMO gap in **4** is 2.55 eV (58.9 kcal mol^{-1}), slightly less than in **2**. During geometry optimization, the Au_4 ring of **4** becomes nearly flat, and the complex assumes almost ideal S_4 symmetry, suggesting that its slight bending in the solid state may result from crystal packing forces. The average Mayer bond order between the gold atoms is 0.19.[14]

Table S1: Crystal Data and Summary of X–ray Data Collection

	**[{KCuA´$_2$}$_2$] (1)**	**[{CuA´}$_4$] (2)**	**[{KAuA´$_2$}$_2$] (3)**	**[{AuA´}$_4$] (4)**
Empirical formula	$C_{18}H_{42}CuKSi_4$	$C_{36}H_{82}Cu_4Si_8$	$C_{18}H_{42}AuKSi_4$	$C_{36}H_{82}Au_4Si_8$
Formula weight	473.51	995.91	606.94	1529.61
Temperature	100.02(10) K	100.00(10)) K	100.3(7) K	100.01(10) K
Wavelength	1.54184 Å	1.54184 Å	1.54184 Å	1.54184 Å
Crystal system	Monoclinic	Monoclinic	Monoclinic	Triclinic
Space group	$P2_1/n$	$I2/a$	$P2_1/n$	$P\overline{1}$
Unit cell dimensions	a = 12.5971(1) Å	a = 18.9909(2) Å	a = 12.44080(10) Å	a = 13.8652(4) Å
	b = 11.0304(1) Å	b = 13.8677(2) Å	b = 11.24230(10) Å	b = 18.5887(5) Å
	c = 20.9947(2) Å	c = 41.2158(4) Å	c = 21.1382(2) Å	c = 23.4070(7) Å
	α = 90°	α = 90°	α = 90°	α = 70.838(2)°
	β = 103.106(1)°	β = 98.2800(10)°	β = 102.8390(10)°	β = 82.884(2)°
	γ = 90°	γ = 90°	γ = 90°	γ = 83.340(2)°
Volume	2841.25(5) Å^3	10741.4(2) Å^3	2882.54(5) Å^3	5636.4(3) Å^3
Z	4	8	4	4
Density (calculated)	1.107 g/cm^3	1.232 g/cm^3	1.399 g/cm^3	1.803 g/cm^3
Absorption coefficient	3.998 mm^{-1}	3.644 mm^{-1}	12.465 mm^{-1}	20.911 mm^{-1}
F(000)	1016	4224	1216	2912
Crystal size (mm^3)	0.15 x 0.13 x 0.1	0.18 x 0.12 x 0.1	0.15 x 0.14 x 0.03	0.22 x 0.16 x 0.1
Crystal color, habit	colorless, block	yellow, block	colorless, block	yellow, block
Theta range for data collection	3.758 to 71.850°	3.3470 to 71.6560°	3.796 to 71.791°	2.525 to 72.042°
Index ranges	$-15 \le h \le 12$, $-13 \le k \le 13$, $-25 \le l \le 25$	$-17 \le h \le 23$, $-16 \le k \le 16$, $-50 \le l \le 50$	$-15 \le h \le 15$, $-10 \le k \le 13$, $-25 \le l \le 25$	$-17 \le h \le 16$, $-20 \le k \le 22$, $-28 \le l \le 28$
Reflections collected	27 269	57 729	27 231	76 630
Independent reflections	5523 [R(int) = 0.0338]	10 408 [R(int) = 0.0359]	5571 [R(int) = 0.0333]	21 641 [R(int) = 0.0466]
Absorption correction	Gaussian	Gaussian	Gaussian	Gaussian
Max. and min. transmission	0.726 to 0.859	0.649 to 1.000	0.263 to 0.850	0.133 to 0.390
Refinement method	Full-matrix least-squares on F^2	Full-matrix least-squares on F^2	Full-matrix least-squares on F^2	Full-matrix least-squares on F^2
Data / restraints / parameters	3380 / 0 / 253	10 408 / 0 / 505	5571 / 0 / 253	21 641 / 48 / 987
Goodness-of-fit on F^2	1.032	1.014	1.073	1.018
Final R indices [I>2sigma(I)]	R_1 = 0.0259, wR_2 = 0.0657	R_1 = 0.0292, wR_2 = 0.0725	R_1 = 0.0234, wR_2 = 0.0586	R_1 = 0.0450, wR_2 = 0.1159
R indices (all data)	R_1 = 0.0287, wR_2 = 0.0678	R_1 = 0.0342, wR_2 = 0.0755	R_1 = 0.0252, wR_2 = 0.0598	R_1 = 0.0515, wR_2 = 0.1213
Largest diff. peak and hole	0.353 and -0.219 e/ Å^{-3}	0.590 and -0.471 e/ Å^{-3}	1.180 and -1.307 e Å^{-3}	2.843 and -1.805 e Å^{-3}

References

[1] F. H. Jardine, L. Rule, A. G. Vohra, *J. Chem. Soc. A: Inorganic, Physical, Theoretical* **1970**, 238–240.

[2] G. A. Bowmaker, C. Di Nicola, C. Pettinari, B. W. Skelton, N. Somers, A. H. White, *Dalton Trans.* **2011**, *40*, 5102–5115.

[3] G. Boche, G. Fraenkel, J. Cabral, K. Harms, N. J. R. van Eikema Hommes, J. Lohrenz, M. Marsch, P. v. R. Schleyer, *J. Am. Chem. Soc.* **1992**, *114*, 1562–1565.

[4] K. T. Quisenberry, J. D. Smith, M. Voehler, D. F. Stec, T. P. Hanusa, W. W. Brennessel, *J. Am. Chem. Soc.* **2005**, *127*, 4376–4387.

[5] O. V. Dolomanov, L. J. Bourhis, R. J. Gildea, J. A. K. Howard, H. Puschmann, *J. Appl. Cryst.* **2009**, *42*, 339–341.

[6] G. M. Sheldrick, *Acta Crystallogr. Sect. A* **2015**, *71*, 3–8.

[7] G. M. Sheldrick, *Acta Crystallogr. Sect. C* **2015**, *71*, 3–8.

[8] M. J. Frisch, G. W. Trucks, H. B. Schlegel, G. E. Scuseria, M. A. Robb, J. R. Cheeseman, G. Scalmani, V. Barone, G. A. Petersson, H. Nakatsuji, X. Li, M. Caricato, A. V. Marenich, J. Bloino, B. G. Janesko, R. Gomperts, B. Mennucci, H. P. Hratchian, J. V. Ortiz, A. F. Izmaylov, J. L. Sonnenberg, Williams, F. Ding, F. Lipparini, F. Egidi, J. Goings, B. Peng, A. Petrone, T. Henderson, D. Ranasinghe, V. G. Zakrzewski, J. Gao, N. Rega, G. Zheng, W. Liang, M. Hada, M. Ehara, K. Toyota, R. Fukuda, J. Hasegawa, M. Ishida, T. Nakajima, Y. Honda, O. Kitao, H. Nakai, T. Vreven, K. Throssell, J. A. Montgomery Jr., J. E. Peralta, F. Ogliaro, M. J. Bearpark, J. J. Heyd, E. N. Brothers, K. N. Kudin, V. N. Staroverov, T. A. Keith, R. Kobayashi, J. Normand, K. Raghavachari, A. P. Rendell, J. C. Burant, S. S. Iyengar, J. Tomasi, M. Cossi, J. M. Millam, M. Klene, C. Adamo, R. Cammi, J. W. Ochterski, R. L. Martin, K. Morokuma, O. Farkas, J. B. Foresman, D. J. Fox, *Gaussian 16 Rev. C.01* **2016**.

[9] A. D. Becke, *J. Chem. Phys.* **1993**, *98*, 5648–5652.

[10] S. Grimme, J. Antony, S. Ehrlich, H. Krieg, *J. Chem. Phys.* **2010**, *132*, 154104/154101–154104/154119.

[11] S. Grimme, S. Ehrlich, L. Goerigk, *J. Comput. Chem.* **2011**, *32*, 1456–1465.

[12] D. Rappoport, F. Furche, *J. Chem. Phys.* **2010**, *133*, 134105.

[13] T. Lu, F. Chen, *J. Comp. Chem.* **2012**, *33*, 580–592.

[14] A. J. Bridgeman, G. Cavigliasso, L. R. Ireland, J. Rothery, *J. Chem. Soc., Dalton Trans*. **2001**, 2095–2108.
